# Leveraging molecular descriptors and explainable machine learning for monomer conversion prediction in photoinduced electron transfer-reversible addition-fragmentation chain transfer polymerization

**DOI:** 10.1038/s41598-025-33553-y

**Published:** 2026-02-09

**Authors:** Berna Alemdag, Azra Kocaarslan, Gözde Kabay

**Affiliations:** 1 Institute of Functional Interfaces (IFG), Department for Bioengineering and Biosystems, Karlsruhe Institute of Technology (KIT), 76344 Eggenstein-Leopoldshafen, Germany; 2 Institute of Nanotechnology (INT), Department for Bioengineering and Biosystems, Karlsruhe Institute of Technology (KIT), 76344 Eggenstein-Leopoldshafen, Germany

**Keywords:** Explainable AI, Molecular descriptors, Polymer informatics, Conversion prediction, SHAP analysis, SMILES, Chemistry, Materials science, Mathematics and computing

## Abstract

This study presents a molecular descriptor-based machine learning (ML) architecture for predicting monomer conversion in photoinduced electron transfer-reversible addition-fragmentation chain transfer (PET-RAFT) polymerization systems. Unlike traditional polymer informatics approaches that treat polymers as single units or use one-hot encoding for reaction components, we decompose each PET-RAFT system into its individual parts: monomer, RAFT agent, and photocatalyst. Next, each element was separately encoded using 2D molecular descriptors derived from SMILES. Using a literature-sourced dataset of 152 PET-RAFT systems, we systematically trained (with fivefold cross-validation, CV) and evaluated 10 ML algorithms. CatBoost showed greater stability across CV-folds (SD = ± 0.07) and was identified as the top performer for monomer conversion prediction (R^2^ = 0.84; RMSE = 10.04 pps; MAE = 8.16 pps). SHapley Additive exPlanations (SHAP) analysis revealed mechanistically interpretable structure–property-performance relationships, highlighting that monomer topological complexity, electronic polarization, and molecular weight together account for over 60% of the model’s predictive power. External validation confirmed CatBoost’s ability to generalize to unseen (meth)acrylates and (meth)acrylamides (MAE = 8.03), with comparable performance to that of the training set. In practice, the learned descriptor-conversion mapping enables fast in silico screening and component ranking, highlighting actionable descriptor ranges and potentially accelerating design-build-test cycles for high-conversion PET-RAFT.

**Supplementary Information:**

The online version contains supplementary material available at 10.1038/s41598-025-33553-y.

## Introduction

Since Staudinger first coined the term “macromolecules” in the 1920 s, research in polymer chemistry has undergone a significant transformation. Traditional statistical and mathematical models, such as Markov chain models developed for copolymerization kinetics^1^ and the Mayo-Lewis equation used to estimate monomer reactivity ratios^2^, have long been instrumental in polymer science. Yet, the persistent demands of sustainable and circular chemistry principles are now driving a paradigm shift toward rational, design-based approaches for polymer discovery^3, 4^

In this evolution, artificial intelligence (AI) tools, materials-genome initiatives, and big data have opened new avenues for data-centric informatics in polymer science and engineering, formalizing the discipline known as “polymer informatics”^5, 6^. The polymer informatics complements traditional theoretical methods and experimental approaches by developing multivariate models to capture quantitative structure–property-performance relationship (QSPPR) patterns within high-dimensional design spaces, utilizing data-driven predictive models, such as machine learning (ML)^7, 8, 9^. However, the widespread adoption of AI/ML-driven methods in polymer informatics is currently restrained due to several factors. Most fundamentally, AI/ML algorithms require curated, high-quality datasets of sufficient size, high fidelity, and chemical diversity to enable robust model training that effectively captures QSPPR patterns^7, 10^. Yet, available data are often sparse, heterogeneous, and experimental results are scattered across various literature sources and proprietary industrial databases, usually employing inconsistent measurement protocols and reporting standards^5, 7, 9^. While open-source initiatives such as the PoLyInfo database^11^ and the Polymer Genome project^12, 13^ have made significant contributions toward democratizing access, they are limited by narrow chemical space coverage, insufficient representation of processing condition effects, and dependence on data combined from diverse experimental methods with varying quality control levels. These fundamental data challenges directly compromise both the reproducibility of individual studies and the generalizability of AI/ML models across diverse polymer systems, hindering the development of truly predictive polymer design frameworks^5, 14^.

Unlike small molecules, polymers are extended macromolecules whose chain length, connectivity, and structural diversity (e.g., linear, branched, cross-linked) introduce numerous features into the design space that can obscure digital encoding. The adoption of the Simplified Molecular-Input Line-Entry System (SMILES) strings from cheminformatics has enabled the encoding of monomers and functional groups in a compact, machine-readable format. SMILES strings are used to generate molecular descriptors, including topological, electronic, and physicochemical indices, via platforms such as RDKit for quantitative structure–activity relationship (QSAR)/QSPPR predictions^15, 16^. However, these encodings often treat polymers as aggregated entities, overlooking the individual contributions of their molecular components, such as monomers, initiators, and catalysts, whose complex interactions strongly impact polymerization performance^17^. Conversely, many existing AI/ML studies represent these components using simple categorical methods, such as one-hot or label encoding, thereby treating each molecule as a separate, interchangeable unit. This approach, which utilizes reaction conditions as features for QSPPR predictions, reduces complex molecular structures to arbitrary labels, thereby failing to harness the rich physicochemical information that governs polymerization behavior^18, 19^. Another critical issue is that many high-performing ML models still operate inherently as black boxes, meaning that they do not explain the reasoning behind a given structure or feature that governs the performance. This lack of mechanistic transparency severely undermines their acceptance as tools for rational polymer design, as chemists require an understanding of QSPPR to trust model predictions and apply them in the wet lab^10^. Approaches such as feature attribution methods, visualization tools, and physics-informed descriptors have started to bridge this gap, allowing models to provide mechanistically meaningful insights^20, 21, 22^. Additionally, attention mechanisms in neural networks can visualize which parts of a polymer structure the model focuses on during performance prediction, providing visual insights into the property prediction of polymers^23^. However, integrating explainability with high predictive accuracy and broad generalization capabilities entails significant trade-offs^21^.

**Fig. 1:**
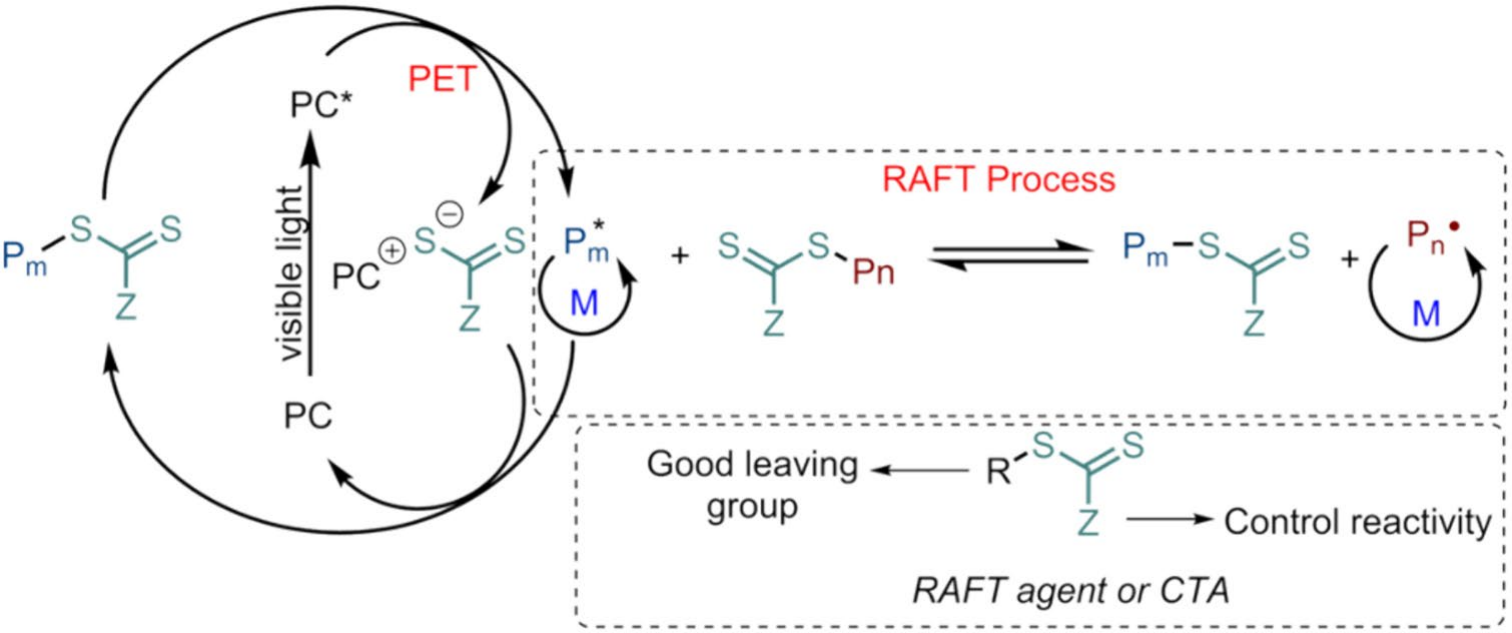
Demonstration of the PET-RAFT mechanism in which light excitation of a photocatalyst initiates the monomer conversion process via photoinduced energy transfer (PET). The excited photocatalyst transfers energy to the RAFT agent or CTA, causing bond cleavage and the formation of radical species that initiate polymerization. Once generated, these radicals enter the RAFT equilibrium, where reversible addition-fragmentation steps control chain growth (P_m_). In this process, propagating radicals add to the CTA, forming an intermediate radical that undergoes fragmentation to regenerate the RAFT agent and release a new radical capable of further propagation (P*_n_). This dynamic exchange between active and dormant chains minimizes irreversible termination, leading to polymers with predictable molecular weights, narrow dispersity, and tunable architectures.

These limitations are pronounced in reversible deactivation radical polymerization (RDRP), also known as living/controlled radical polymerization (LRP/CRP), because complex equilibrium dynamics govern the polymerization mechanism among activation, deactivation, and chain-transfer events rather than by simple linear structure–property correlations. These controlled radical polymerization approaches enable the synthesis of polymeric architectures that exhibit predictable molecular weight, low molar mass dispersity (Đ), high end-group fidelity, and the capacity for continued chain growth^24^. RDRP operates through a dynamic equilibrium between active and dormant polymer chains. This equilibrium can be established via two main mechanisms: (i) *reversible deactivation*, which relies on the persistent radical effect and is exemplified by nitroxide-mediated polymerization (NMP) and metal-mediated living radical polymerization/atom transfer radical polymerization (LRP/ATRP); or (ii) *degenerative transfer*, as in Reversible Addition-Fragmentation Chain Transfer (RAFT) polymerization. In degenerative transfer systems, the overall number of radicals remains constant during activation-deactivation events, meaning that an external radical source (commonly a radical initiator) is still required^25^. Among these controlled polymerization strategies, RAFT and its photoinduced variant, Photoinduced Electron/Energy Transfer-RAFT (PET-RAFT) polymerization, are particularly attractive due to their broad monomer compatibility and their ability to achieve spatiotemporal control over chain growth, thereby enabling the rational design of complex macromolecular architectures. However, polymerization outcomes are determined by the complex, nonlinear interactions among multiple reactants, including the monomer, the RAFT agent (also known as a chain transfer agent, CTA), the photocatalyst, and reaction conditions (e.g., temperature, pH, solvent, and oxygen). Figure 1 illustrates the PET-RAFT mechanism, highlighting the key species involved in the polymerization process. Current mechanistic studies show that the choice of photocatalyst, monomer, and RAFT agent directly influences the degree of control achieved during polymerization^26^.

While traditional kinetic models, combined with computational simulations, are informative to a certain extent in understanding structure–reactivity relationships, they are time- and resource-constrained, with significant limitations in capturing multivariate interactions, and current applications are restricted to specific polymer classes^27, 28^. Black-box ML approaches, although many have established high predictive accuracies for the QSPPR prediction tasks, most offer little or no mechanistic explanation for the observed trends, making it extremely difficult to quantify individual component contributions or understand how a specific modification in one component (e.g., changing a RAFT agent’s Z-group) might affect overall polymerization performance^10, 29^.

**Fig. 2:**
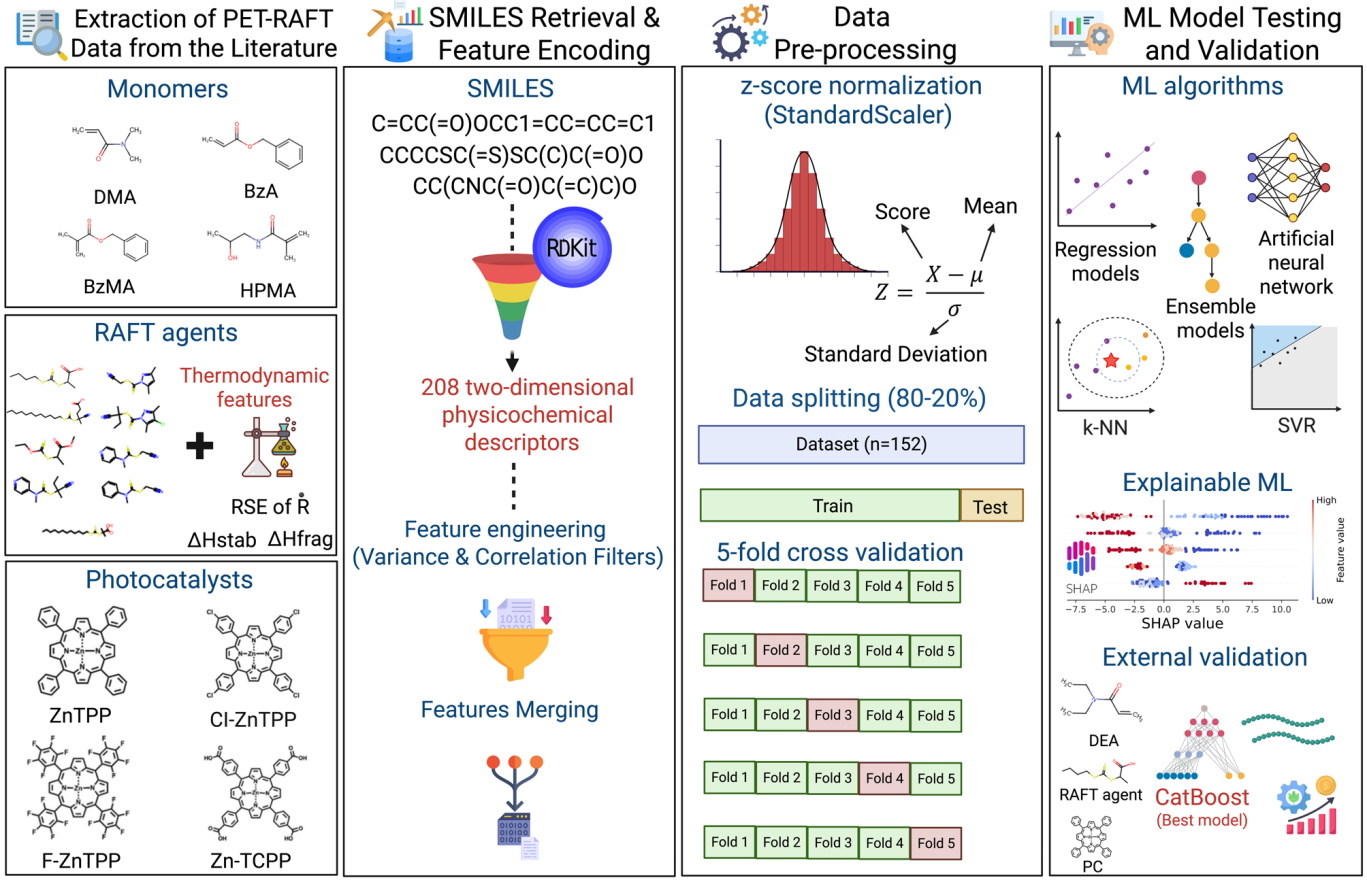
Workflow of the ML pipeline for predicting monomer conversion in PET-RAFT polymerization reaction. Monomers, RAFT agents, and photocatalysts were encoded as SMILES and transformed into 208 two-dimensional physicochemical descriptors using RDKit. After feature engineering (variance- and correlation-based filtering) and feature merging, the descriptors were standardized using z-score normalization. The dataset (n = 152) was split into 80/20 training/test splits and evaluated using fivefold cross-validation across multiple ML algorithms. Following the selection of CatBoost as the best-performing model, explainable ML techniques (global and local SHAP analyses) identified key molecular descriptors and their relative impact on the model’s predictions, along with mechanistic insights. External validation with structurally distinct, unseen monomers confirmed the model’s generalizability. The figure is created in Biorender.com under a paid academic subscription (https://BioRender.com/iiu1cvr).

We present an explainable, descriptor-based ML model that predicts monomer conversion in PET-RAFT, maps molecular descriptors to conversion, quantifies the contributions of the monomer/RAFT agent/photocatalyst, and guides component design and selection for high-conversion formulations. While we recognize that polymerization success is influenced by both molecular structure and reaction conditions, this study intentionally focuses solely on intrinsic molecular descriptors to uncover QSPPR patterns independent of experimental variables (i.e., pH, temperature, light intensity). Therefore, each reactant, including the monomer, RAFT agent, and photocatalyst, is encoded using SMILES strings and RDKit-derived 2D descriptors, which are complemented with thermodynamic parameters. By training our model solely on molecular features using a high-throughput PET-RAFT dataset (n = 152) generated under standardized conditions, we achieved a good prediction accuracy (R^2^ = 0.84) with the CatBoost regressor. This was further supported by mechanistic insights from SHAP analysis, which identified key molecular features within each component that influence monomer conversion. Although some limitations remain, such as neglecting solvent effects and the need for validation across broader chemical spaces, this approach offers a new perspective on polymer informatics by linking molecular descriptors to polymerization performance, even with limited data (Fig. 2).

## Results and discussion

### SMILES-based multi-component monomer conversion prediction architecture

The experimental dataset was sourced from the Boyer team, who performed high-throughput screening of 152 PET-RAFT polymerization reactions under standardized conditions (λ_max_ = 530 nm, 5.5 mW cm^−2^)^30^. The histogram in Figure S1 indicates that monomer conversion (%) values in the dataset range from approximately 4% to 98%, with a mean of 47.93% and a standard deviation (± SD) of 29.27% (Table S1). This provides a homogenous distribution of the target variable within the dataset, a critical requirement for reliable and robust model training.

**Fig. 3:**
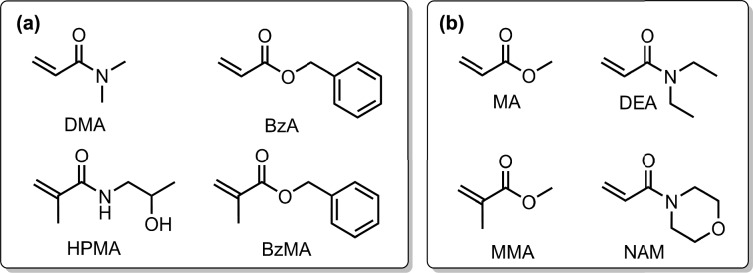
Chemical structures of monomers used for ML model development. (a) Training set: DMA, BzA, HPMA, and BzMA. (b) External validation set MA, DEA, MMA, and NAM. The monomers within the training set were selected to encompass a diverse range of steric and electronic environments during model development. In contrast, the external validation set included unseen but structurally related monomers to those in the training set, enabling the evaluation of the model’s ability to generalize beyond the known monomers.

The training set consists of four types of monomers: N, N-dimethylacrylamide (DMA), benzyl acrylate (BzA), N-(2-hydroxypropyl)acrylamide (HPMA), and benzyl methacrylate (BzMA), which were chosen to represent a diverse range of physicochemical features relevant to PET-RAFT polymerization (Fig. 3a). These monomers include (meth)acrylamides (DMA, HPMA) and (meth)acrylates (BzA, BzMA), thereby covering different substitution patterns at the vinyl group as well as varying steric and electronic environments (e.g., electron-donating amideand bulky benzyl substituents). This diversity provides the model with a chemically informative basis for learning key QSPPRs that were once hidden in the data. For external validation, we selected monomers outside of the training set, methyl acrylate (MA), diethyl acrylamide (DEA), methyl methacrylate (MMA), and N-methylacrylamide (NAM), that belong to the same broader (meth)acrylate and (meth)acrylamide families but differ in size, polarity, and substitution pattern (Fig. 3b). Using structurally related yet distinct monomers allowed us to assess the model’s generalization ability, i.e., its capacity to transfer learned QSPPR to make accurate predictions on unseen yet chemically relevant systems without memorization. The chemical structures of RAFT agents and photocatalysts are shown in Figure S2.

Our main methodological innovation lies in the systematic decomposition and separate encoding of each reaction component, namely monomer, RAFT agent, and photocatalyst, using SMILES-derived molecular descriptors extracted via RDKit, supported by thermodynamic parameters of the RAFT agents (i.e., the radical stabilization energy, the fragmentation efficiency, and enthalpy values of RAFT stability). Unlike most approaches, which either combine all components into a single polymer representation or rely on simple one-hot encoding, treating molecules as categorical variables, our method preserves the chemical identity and physicochemical properties of each component separately. This decomposition strategy aimed to provide the model with the ability to learn cross-component interactions, for instance, how a specific RAFT agent’s fragmentation enthalpy influences conversion differently depending on the monomer’s steric bulk or the photocatalyst’s redox potential^24^.

Initial feature extraction yielded 208 features encompassing various 2D molecular descriptors (e.g., surface area, topological, and electronic descriptors) for each PET-RAFT component. The number of descriptors was systematically reduced through variance filtering (threshold < 0.05) and correlation analysis (|r|> 0.95) to eliminate redundant features, thereby preserving collinearity while maintaining computational power and minimizing noise^[31]^.After dimensionality reduction, the final feature set comprised 83 descriptors: 57 SMILES-derived molecular descriptors and 3 thermodynamic parameters for RAFT agents, 8 monomer descriptors, and 15 descriptors for photocatalysts (Table S2). The monomer conversion percentage (yield) was designated as the target variable to be predicted.

### Model training and stability assessment

We systematically evaluated ten diverse ML algorithms to assess their capability for predicting monomer conversion percentages using SMILES-derived descriptors **(**Table S2 and S3**)**. The complete dataset (n = 152) was partitioned into an 80/20 training/test split. fivefold cross-validation (CV) was used to train and assess the stability of the ML models. The held-out set was used to evaluate each ML model’s final predictive performance via internal validation. It is essential to note that, although not extensively used, performing *k-fold* CV maximizes the usage of available data, as each data point within the given dataset is randomly assigned to both training and internal validation sets across folds. Therefore, it is critical to ensure model robustness and mitigate the risk of overfitting, particularly for small datasets such as the one used in this study^31^.

**Fig. 4:**
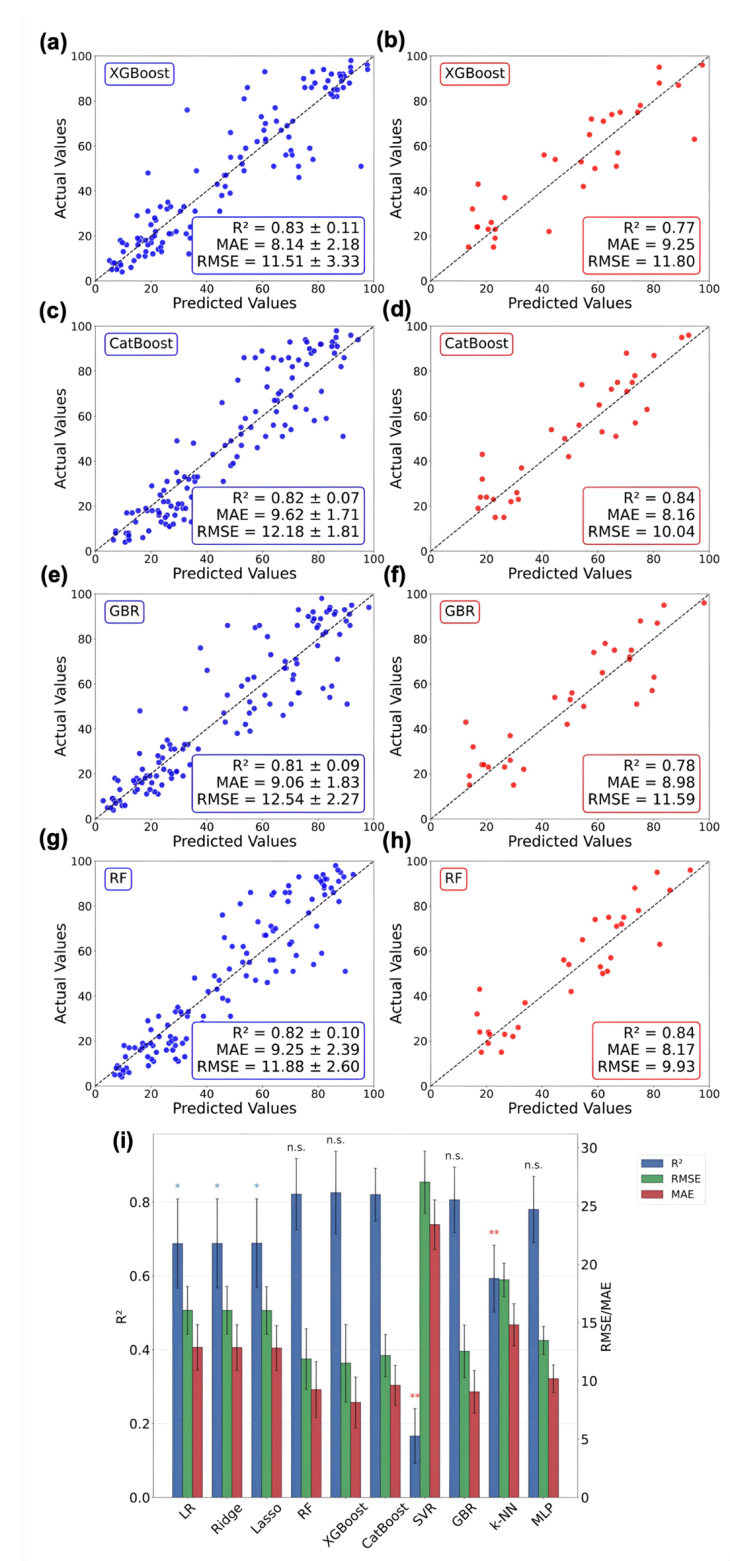
Parity plots demonstrating predicted versus actual monomer conversion percentages for (a, b) CatBoost, (c, d) RF, (e, f) GBR, and (g, h) XGBoost. The left panels show predictions from the fivefold CV-assisted model training process. In contrast, the right panels correspond to predictions made using the held-out test set to evaluate the performance of the ML algorithms. The dashed diagonal line (x = y) represents ideal prediction (predicted = actual). (i) CV performance analyses of ML models obtained during model training, with statistical comparison to CatBoost. The bar chart shows the average R^2^, RMSE, and MAE obtained from fivefold CV on the training dataset. Error bars represent the ± SD across CV folds. RMSE and MAE units are in pps. Statistical significance of the difference in R^2^ between each model and CatBoost was evaluated using a paired t-test (**p < 0.01, **p < 0.05;* p* > 0.05, *n.s*. = not significant).

The parity plots (Figs. 4a, 4c, 4e, 4** g**, and Figures S3**a, S3c, S3e, S3g, S3i, S3k**) across various algorithms show the distribution of predicted versus actual monomer-conversion data. The diagonal line (x = y) indicates where predicted monomer conversion percentages match the actual values across the entire conversion range (~ 4–98%). The training/test performance evaluation of the ten models was conducted using multiple metrics: the coefficient of determination (R^2^) for prediction accuracy, root mean square error (RMSE), and mean absolute error (MAE) for error assessment. Meanwhile, the ± SDs of the R^2^ values were calculated to assess the relative stability of the models across CV folds, with lower variance indicating greater reliability and robustness (Fig. 4i**, **Table S4). Because monomer conversion is reported as a percentage, the RMSE and MAE, which quantify the residuals between predicted and actual results, are expressed in percentage points (pps).

During model training, all four tree-based ensemble methods demonstrated strong predictive capability with tight clustering around the ideal prediction line. This can be linked to the effectiveness of these methods in managing hierarchical decisions that capture non-linear, multi-component interactions through recursive data partitioning and feature splitting. The eXtreme Gradient Boosting (XGBoost) algorithm **(**Fig. 4a**)**, achieved the highest average R^2^ across CV folds (0.83 ± 0.11), followed closely by the categorical boosting or CatBoost **(**Fig. 4c**)** algorithm (R^2^ = 0.82 ± 0.07), the Random Forest algorithm or RF (R^2^ = 0.82 ± 0.10), and the Gradient Boosting regressor or GBR (R^2^ = 0.81 ± 0.09), all displaying a small scattering around the diagonal line (Figs. 4g, and 4e**)**. The comparative analysis of error metrics among the trained ensemble algorithms reveals a clear performance hierarchy (Fig. 4i).

XGBoost **(**Fig. 4a**)** recorded the lowest RMSE of 11.51 ± 3.33 pps and the lowest MAE of 8.14 ± 2.18 pps, suggesting comparable performance in minimizing absolute deviations. This performance was achieved through XGBoost’s gradient-boosted decision trees, which incorporate built-in regularization to prevent overfitting^32^. RF exhibited a slightly higher RMSE (11.88 ± 2.60 pps) but a low MAE (9.25 ± 2.39 pps), likely due to the multiple decision trees built on bootstrapped datasets (Fig. 4g)^33^. GBR **(**Fig. 4e**)**, on the other hand, performed poorly amongst all ensemble methods (RMSE = 12.54 ± 2.27 pps and MAE = 9.06 ± 1.83 pps), likely due to its sequential boosting approach being more susceptible to overfitting on small datasets compared to the more sophisticated regularization mechanisms in XGBoost and CatBoost. Notably, CatBoost, despite not leading in accuracy or error metrics (RMSE = 12.18 ± 1.81 pps and MAE = 9.62 ± 1.71 pps), showed the slightest deviation in R^2^ (± 0.07) across the CV folds, suggesting a robust model. The CatBoost model’s robustness, paired with its prediction accuracy of 0.82, arises from its capacity to avoid overfitting via ordered boosting and symmetric tree structures. This makes it particularly well-suited to handling the variety of descriptor types frequently encountered in chemical systems^34^.

Other algorithms exhibited systematic limitations in monomer-conversion prediction (Figure S3**, **Table S4). The Multi-layer Perceptron (MLP) algorithm demonstrated moderate performance, with an R^2^ of 0.78 ± 0.09, an RMSE of approximately 14 pps, and an MAE of approximately 11 pps, respectively (Figure S3**.g)**. It is believed that MLP’s performance was limited by the difficulty of training neural networks on relatively small datasets. Linear and regularized regression models (linear regression or LR, Ridge, and Lasso) clustered around an R^2^ of 0.69, with significantly higher RMSE (~ 16.0 pps) and MAE (~ 12.8 pps), indicating moderate predictive performance **(**Figures S3**.a, c, and e)**. These approaches are inherently limited in capturing non-linear PET-RAFT structure-performance relationships, as indicated by increased data scatter in the parity plots, especially at higher conversion values (greater than 50%). The instance-based k-Nearest Neighbors (k-NN) regressor showed slightly lower accuracy with an R^2^ of 0.59 ± 0.09 (RMSE = 18.67 ± 1.44 pps; MAE = 14.81 ± 1.81 pps), because local similarity in molecular descriptor space does not reliably translate to similar conversion outcomes in complex multi-component systems **(**Figure S3**.i)**^35^.Finally, the Support Vector Regressor (SVR), although being an algorithm capable of modeling both linear and non-linear relationships by mapping data to a higher-dimensional hyperplane using kernel methods, in our specific case, it exhibited the weakest overall performance and the highest error margins, recorded at R^2^ of 0.17 ± 0.07 and RMSE and MAE around 27 pps and 23 pps, respectively **(**Figure S3**.k)**. This suggests that radial basis function kernels are insufficient for capturing complex distance-based relationships in multi-component SMILES-derived features, as previously noted^18^.

Next, we evaluated the average predictive accuracy and stability of all ML models across validation folds (Figure S4). Among ensemble models, CatBoost exhibited the lowest fold-to-fold variability (SD = 0.07) and achieved competitive accuracy (0.82), whereas XGBoost and RF achieved similar accuracy but showed greater variability (± 0.10–0.11). Afterward, statistical analysis using a paired *t*-test on the R^2^ values from each cross-validated model compared to those of CatBoost (Fig. 4i) revealed that CatBoost’s CV performance was significantly better (p** < 0.01) than k-NN and SVR, as well as all linear models: LR, Ridge, and Lasso (p* ≤ 0.05). Conversely, the CV performance of XGBoost, RF, GBR, and MLP did not significantly differ from that of CatBoost (*n.s., p* > 0.05), indicating that these algorithms performed similarly during model training. These results confirm that ensemble methods based on decision trees achieved higher accuracy, with CatBoost offering the best balance between performance and stability during model training.

### Model performance evaluation

The tree-based ensemble methods (e.g., CatBoost, RF, GB, and XGBoost) outperform all other models in monomer conversion prediction. The top-performing algorithm is CatBoost (Fig. 4d), which achieved an R^2^ of 0.84 on the unseen test set, accounting for 84% of the variance, and closely matches its CV performance (R^2^ = 0.82), indicating consistent predictive performance without overfitting. The RMSE value indicates that predictions typically deviate from actual conversion values by approximately 10 pps, with larger deviations being weighted more heavily due to the squaring operation. In comparison, the MAE shows that, on average, predicted conversion values differ from actual values by 8.16 pps when all errors are weighted equally. Other ensemble models also perform strongly: RF (R^2^ = 0.84, Fig. 4h), GBR (R^2^ = 0.78, Fig. 4f), and XGBoost (R^2^ = 0.77, Fig. 4b) all reached an R^2^ around 0.80 with RMSE and MAE varying between 9.93–11.80 pps and 8.17–9.25 pps, respectively. On the other hand, the lower average accuracy (R^2^ = 0.66) and higher prediction errors (RMSE ~ 14.4 pps, MAE ~ 11.3 pps) observed with linear regression models indicate, once again, their failure to capture the complex, nonlinear relationships in PET-RAFT systems (Table S5).

The parity plots (Figs. 4b, 4 d, 4f, and 4 h and Figures S3b**, 3 d, 3f., 3 h, 3j, and 3 l**) provide complementary visual insight into the performance evaluation, with the CatBoost model displaying the tightest clustering around the diagonal line, with minimal scatter across the entire conversion range. Other tree-based ensemble methods, such as RF, GBR, and XGBoost, exhibit similar patterns of strong predictive accuracy, albeit with slightly greater scatter than CatBoost. Notably, these models maintain consistent performance across both low and high conversion regimes, indicating comprehensive prediction capability of CatBoost across diverse monomer-RAFT agent-photocatalyst combinations.

Other models reveal systematic limitations that demonstrate their inability to accurately capture the complex chemical relationships among multiple reacting components. Both the k-NN regressor and MLP algorithms exhibited intermediate performance (R^2^ ~ 0.70, RMSE ~ 13.6 pps, MAE ~ 10.7 pps), with greater scatter across the conversion range than the tree-based methods (Figures S3**.j and h**). Conversely, linear regression methods like LR, Ridge, and Lasso exhibited notable scattering, especially at higher conversion values (< 50%), with SVR performing the poorest (R^2^ = 0.16, RMSE = 22.7 pps, MAE = 18.74 pps), as shown by the spread around the ideal prediction line (Figure S3**.b, d, f, and l**).

We observe that the superior performance of ensemble trees aligns with recent chemoinformatic benchmarks, which indicate that non-linear models (particularly decision-tree-based and kernel methods) frequently outperform linear models in predicting QSPPRs^7, 36^. Furthermore, the performance results confirm that SMILES-based descriptors require algorithms capable of modeling multi-modal component interactions for accurate prediction of monomer conversion within the complex chemical system. The previously explained analyses demonstrate the training/test performance and reliability of the ML models through internal validation within the studied parameter space. Yet, it does not reflect their generalizability to other PET-RAFT systems. To expand this, we performed a subsequent “external” validation using a small dataset sourced from literature^37, 38^, with predictions made solely using the CatBoost model due to its statistically confirmed superior stability and testing performance^39^.

### Explainable machine learning by SHAP analyses

**Fig. 5:**
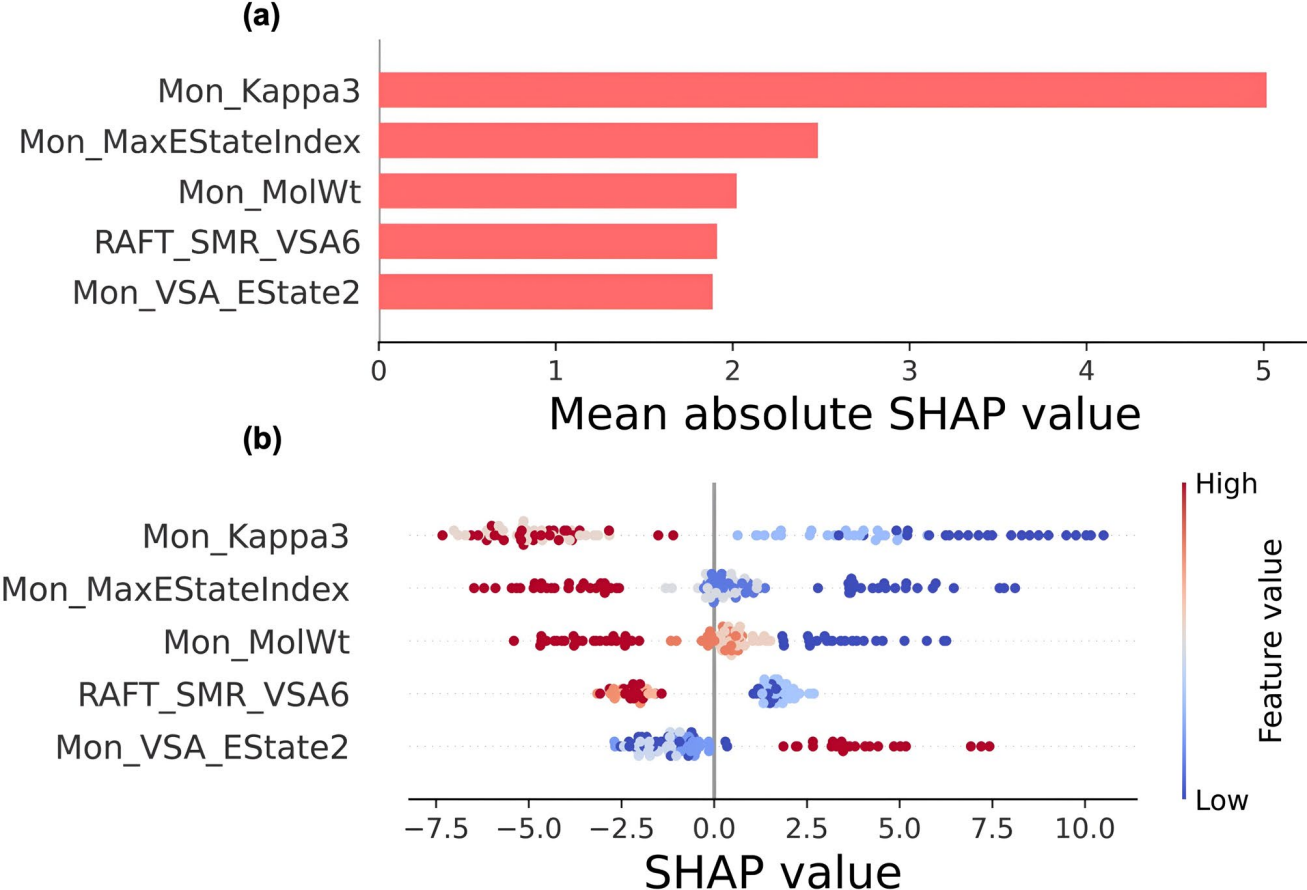
Global feature impact ranking of the top eight molecular descriptors on monomer conversion prediction. (**a**) The bar chart shows the magnitude of each feature’s contribution to the individual prediction, ranked by its assigned mean absolute SHAP value across all samples in the dataset. (**b**) The beeswarm plot displays the impact of the same descriptors on the monomer conversion prediction. Each point represents a single experiment, colored by the raw feature’s SHAP value, computed based on the impact of all other features on monomer conversion prediction. Blue (low values) indicates that a feature has a negative influence on the predicted outcome, whereas red (high values) indicates a positive impact. The vertical line at x = 0 indicates no effect, with the vertical spread reflecting variability across samples rather than a single local explanation.

To clarify the mechanistic drivers of monomer conversion prediction, we utilized SHAP (SHapley Additive exPlanations) analyses on CatBoost predictions. Figure 5 presents a SHAP summary plot for the top five features ranked by their corresponding global importance, along with a beeswarm plot illustrating the directionality of each molecular descriptor on CatBoost’s monomer conversion prediction. For a complete list of global and local SHAP analyses, please refer to Figures S5 and S6 in the *Supporting Document*.

Global feature importance analysis, as assessed by SHAP plots, revealed a clear hierarchical structure in the influence of molecular descriptors on monomer conversion predictions. Mon_Kappa3 showed the highest overall importance, with a mean absolute SHAP value that was almost twice that of Mon_MaxEStateIndex, followed by Mon_MolWt, RAFT_SMR_VSA6, and Mon_VSA_EState2. Below, we provide a thorough explanation of the SHAP plots for our dataset, supported by mechanistic insights from established PET-RAFT literature.

#### Mon_Kappa3

The third-order Kier κ shape index, Mon_Kappa3 or κ_3_, is the strongest predictor of monomer conversion, with the highest mean absolute SHAP value of 5.01, indicating a strong negative correlation with monomer conversion. This topological index relates to branching, cyclicity, and shape complexity through three-bond patterns^40^. In this study, κ_3_ likely correlates with pendant-group complexity rather than backbone branching, as all studied monomers, including DMA, BzA, BzMA, and HPMA, share linear α, β-unsaturated structures typical of acrylate monomers. The SHAP value distribution indicates that low κ₃ values are associated with higher conversion rates, whereas high κ₃ values tend to reduce conversion rates.

The pendant group topology affects radical stabilization and RAFT dynamics via (i) steric effects, (ii) conformational flexibility, and (iii) electronic delocalization^27^. (i) Bulky R groups can induce unfavorable steric interactions, affecting the stability of the RAFT agent, the RAFT-adduct radical, and the efficiency of fragmentation and addition steps. Steric hindrance at the carbon center of the C = S bond, for instance, makes addition to carbon kinetically less favorable than addition to sulfur. This is because the spin density is considerably greater on sulfur than on carbon in the triplet configuration of the thiocarbonyl, leading to a much stronger early bonding interaction when addition occurs at sulfur^27^. This steric obstruction directly reduces degenerative chain-transfer rates, accounting for the observed mean absolute SHAP range of −0.8 to −7.3 for high-κ₃ monomers. (ii) Conformational flexibility or rigidity around the radical center is critical for the accessibility to the thiocarbonylthio group^41^. Lower κ_3_ monomers, such as DMA (κ_3_ = 1.98), are more conformationally flexible around the radical center, which, in turn, improves accessibility to the thiocarbonylthio group, resulting in higher conversion (~ 69% on average). This enhanced conformational sampling accelerates the RAFT equilibrium, thereby improving conversion by + 4 to + 10 pps relative to our SHAP analysis. Aromatic pendant groups in BzA and BzMA with κ₃ of 2.58 and 3.11 stabilize radicals through benzylic resonance, reducing reactivity toward both propagation (propagation rate constant, *k*_*p*_) and RAFT chain transfer “addition” to the C = S (addition rate constant, *k*_*add*_)^42^. The combined stabilization and steric effects surpass any reduction in termination, so high-κ₃ monomers show lower conversions under the same conditions (BzA/BzMA typically around 53%/29% on average) than low-κ₃ DMA (about 69% on average). HPMA (κ₃ = 4.08), the highest-κ₃ monomer in the dataset, lacks benzylic resonance yet exhibits a strong adverse κ₃ effect, driven predominantly by steric and conformational restrictions that can reduce the leaving-group ability of its propagating radical, resulting in an average monomer conversion of 42%. Overall, κ₃ acts as a concise proxy for pendant complexity, surpassing the effective growth/RAFT turnover rate. The detailed SHAP analysis, showing an approximately 18-unit difference for the Mon_Kappa3 descriptor reported here, along with mechanistic explanations from the literature, confirms that reducing pendant complexity enhances PET-RAFT efficiency, explaining the industry’s preference for simpler acrylates and acrylamides in large-scale applications^24, 43^.

#### Mon_Max_EState index

Mon_MaxEStateIndex, defined as the highest electrotopological state index value among all atoms in the monomer, measures the electronic environment of atoms by considering both their intrinsic electronic state and influences from neighboring atoms^44^. In the global importance plot, it ranks second for the monomer conversion prediction with a mean absolute SHAP value of 2.5 (Fig. 5a). The electron-donating nature of substituents influences the polymerization rate, as electron-donating groups stabilize the radical intermediate and lower the activation barrier for propagation. Consistent with this principle, the beeswarm plot (Fig. 5b) indicates that low MaxEStateIndex values, corresponding to less polarized environments, are associated with higher monomer conversion. This reflects the well-known trend that electron-donating substituents activate the monomer double bond, making it more reactive toward polymer radicals and thereby increasing propagation rates^45, 46^. In our dataset, DMA with delocalized amide resonance, whose delocalized electron density across the C = O-N bond (lowest MaxEStateIndex ~ 10.32), exhibits the highest monomer conversion performance, reaching 68.6% (12–98% range). HPMA with its hydroxyl group creates localized polarity at the alcohol carbon (high MaxEStateIndex ~ 10.38), achieves moderate conversions averaging 42.0% (15–81% range). On the other hand, BzA exhibits an intermediate MaxEStateIndex (~ 10.64) with a moderate average conversion of 53.5% (9–95% range). BzMA, with the highest MaxEStateIndex (~ 10.99), due to the polarized ester-benzyl environment, exhibits the poorest performance, with an average conversion of 29.3% (4–70% range). The SHAP analysis correctly shows a homogeneous distribution of high MaxEStateIndex values, indicating that higher electronic polarization consistently impedes polymerization under identical experimental conditions.

#### Mon_MolWt

The monomer molecular weight (Mon_MolWt) descriptor showed the third-highest impact in our SHAP analysis. We interpret this as a proxy for two primary physicochemical effects. First, a monomer’s size and steric bulk directly influence the propagation rate constant (*k*_*p*_)*,* where larger substituents can introduce steric hindrance around the reactive double bond, potentially elevating the activation barrier for addition to the propagating radical chain end^47, 48, 49^.

Second, and more critically in the context of diffusion, a monomer’s mass is related to its hydrodynamic volume, which, in turn, influences the viscosity of the reaction environment. In other words, as polymerization proceeds, the system’s viscosity increases. It is well established in the previous literature that, in free-radical and controlled radical polymerizations, bimolecular termination between growing macroradicals becomes diffusion-controlled at moderate to high conversions, a phenomenon known as the Trommsdorff-Norrish effect^50, 51^. The termination rate coefficient (*k*_*t*_) exhibits a strong dependence on the chain length of the reacting radicals and decreases significantly under these conditions^52, 53^.

Thus, rather than reflecting segmental diffusion of monomers, the Mon_MolWt descriptor in our model likely captures how monomer structure indirectly shapes the evolving transport environment of the polymerizing system. This interpretation aligns with our SHAP value distribution and with established principles of radical polymerization kinetics^51, 53, 54^.

#### RAFT_SMR_VSA6

The RAFT_SMR_VSA6 represents the molecular refractivity sum for atoms with van der Waals surface area (VSA) in the sixth bin, capturing polarizability contributions from medium-sized molecular fragments within the RAFT agent. In the global feature-importance plot, it ranks fourth for monomer conversion prediction, with a mean absolute SHAP value of 1.9 (Fig. 5a). The beeswarm plot (Fig. 5b) indicates primarily positive effects for high SMR_VSA6 values, which range from + 1 to + 3, and a negative impact for low values, spanning from −2.8 to −1.25.

In RAFT polymerization, the Z-group’s electronic and steric properties critically influence the radical stabilization and chain transfer efficiency. The degree of control achievable in RAFT relies on the propensity for the propagating radical to add to the C = S bond and the subsequent ability of the propagating radical to be released from the RAFT-adduct radical, both of which are dependent on the steric and electronic properties of the R (leaving) and Z (activating) groups^27, 55^. Previous literature suggests that electron-withdrawing substituents on aromatic Z-groups enhance chain transfer coefficients and improve polymerization control, while electron-donating groups have the opposite effect^56^. For RAFT-adduct radicals, stability significantly increases if Z is a π-acceptor group, like cyano or phenyl groups. This stabilization is common for carbon-centered radicals and can be enhanced by captodative effects from SR groups. We observe that dithiobenzoate RAFT agents with aromatic Z-groups show higher SMR_VSA6 values (~ 8.5–9.2), potentially due to the phenyl ring’s substantial polarizability contribution, consistent with their greater polarizability from π-electron systems^30^. This correlates with generally higher conversions (45–95%) compared to aliphatic dithiocarbonates with lower SMR_VSA6 values (conversions of 20–65%), aligning with established RAFT principles where aromatic Z-groups provide superior radical stabilization.

#### Mon_VSA_EState2

Mon_VSA_EState2, defined as the VSA of atoms with electrotopological state indices in the second bin, captures electron-rich surface regions accessible for intermolecular interactions^57^. Our data indicate a mean absolute SHAP value of about 2.5, showing complex, context-dependent behavior. The beeswarm plot reveals that both high and low values of VSA_EState2 can influence monomer conversion either positively or negatively. According to RDKit values, DMA’s extensive amide resonance through two N-methyl groups is expected to yield a high VSA_EState2, which generally correlates with positive SHAP contributions, along with its low κ₃ and molecular weight. However, BzA and BzMA show slightly lower VSA_EState2 values, which may reflect less favorable electronic surface distributions. Together with the steric bulk of the benzyl substituent, these effects likely contribute to their reduced polymerization performance. This context dependence explains the scattered SHAP distribution and underscores the importance of considering descriptor interactions.

### Local explanations

To complement global interpretations and understand the mechanistic drivers at both ends of the polymerization efficiency spectrum, we selected extreme cases from our conversion distribution (the samples with the lowest (4.0%) and highest (96.0%) experimentally determined monomer conversion percentages). We used SHAP waterfall plots for local interpretation of each molecular descriptor’s impact on the CatBoost regressor’s prediction.

**Fig. 6:**
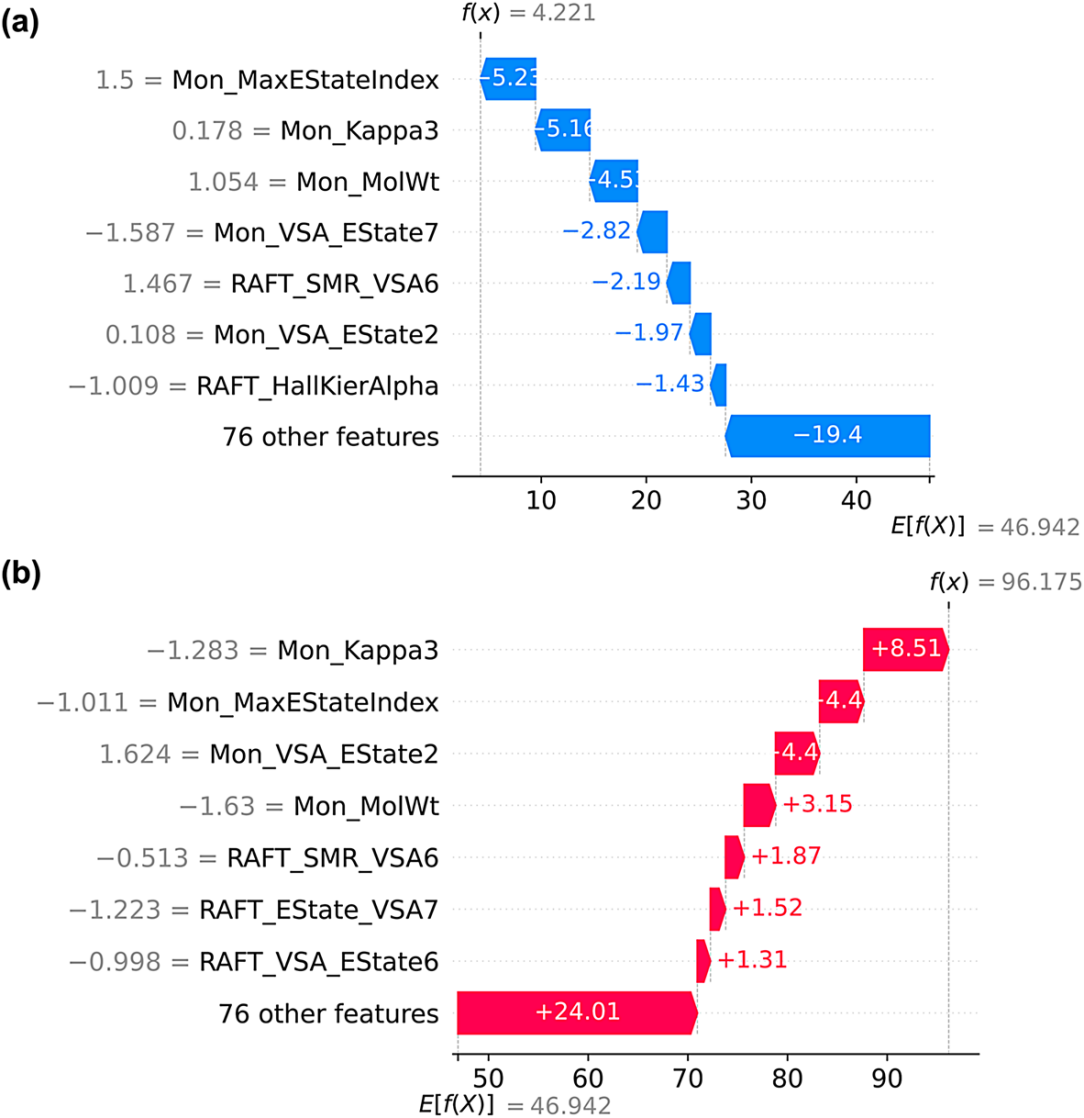
Local SHAP waterfall explanations for monomer conversion. The local attributions of molecular descriptors’ individual impact on the test samples with the (**a**) lowest (~ 4%) and (**b**) highest (~ 96%) monomer conversion values. Each plot displays the local attributions of molecular descriptors for individual experiments; the average monomer conversion*, E[f(X)]*, combined with all molecular descriptor contributions, accounts for the model prediction. Red bars show increases, while blue bars indicate decreases in the predicted conversion percentages.

Figure 6a presents the SHAP waterfall plot for the lowest monomer conversion (4% in the experimental dataset), demonstrating how unfavorable features collectively suppress conversion to a level far below the average monomer conversion of 46.94%. The model’s prediction of 4.22%, which deviates from the experimentally determined conversion by approximately 0.2 pps, confirms its accuracy.

The dominant negative contributor is Mon_MaxEStateIndex (−5.23 pps), indicating this sample contains a monomer with highly polarized electronic centers that create unfavorable radical stabilization. Benzyl (meth)acrylate esters exhibit distinctive kinetics; for benzyl acrylate, the measured interchain chain-transfer constants are low (∼10^–4^ at 100–120 °C), indicating reduced interchain transfer. This behavior is consistent with resonance stabilization in benzylic environments and with reported solvent-dependent *k*_*p*_ for benzyl methacrylate^58, 59, 60^. The substantial Mon_Kappa3 penalty (−5.16 pps) confirms the presence of a topologically complex monomer: the aromatic pendant groups characteristic of our benzyl-containing monomers that sterically hinder the approach to the RAFT agent. Mon_MolWt contributes −4.5 pps, identifying this as one of the heavier monomers (either BzMA or BzA at 176 g mol^−1^ and 162 g mol^−1^), where diffusion limitations severely restrict radical encounters. The Mon_VSA_EState7 penalty (−2.82 pps) further supports the presence of extensive sp^2^-hybridized aromatic carbon environments that stabilize radicals via resonance, thereby reducing their reactivity in chain-transfer processes. RAFT agent descriptors also contribute negatively: RAFT_SMR_VSA6 (−2.19 pps) suggests poor polarizability matching, while RAFT_HallKierAlpha (−1.43 pps) indicates suboptimal molecular connectivity for this monomer-RAFT combination.

The Mon_VSA_EState2 contribution (−1.97 pps) reveals inadequate electron-rich surface area for favorable solvent interactions, likely due to electron density localization in the aromatic ring rather than being distributed across the molecule. The cumulative effect of 76 additional features contributes −19.4 pps, demonstrating that multiple minor unfavorable interactions compound to create poor polymerization conditions, resulting in a total monomer conversion of 4.22%. This sample indeed corresponds to the BzMA-DTC2 monomer-RAFT agent combination, confirming the mechanistic insight derived from the waterfall graph, where steric hindrance, electronic mismatch, and poor RAFT agent compatibility collectively suppress conversion to near-zero levels.

On the other hand, Fig. 6b illustrates the SHAP waterfall plot for the best-performing polymerization reaction, showing how favorable molecular features synergistically enhance monomer conversion from the baseline level of 49.94% to 96.2%. The model’s exceptional accuracy (0.2% error in comparison to actual monomer conversion value) demonstrates its reliability for optimal polymerization conditions. The most significant positive contribution comes from Mon_Kappa3 (+ 8.51 pps), indicating this sample contains DMA with its simple dimethylamide pendant group (low κ₃). This minimal topological complexity may facilitate unhindered contact with the RAFT agent, thereby maximizing chain-transfer efficiency. Mon_MaxEStateIndex contributes + 4.4 pps, confirming the resonance delocalization in DMA`s amide group supports consistent propagation reactivity. Mon_VSA_EState2 also adds + 4.4 pps, reflecting DMA’s favorable electron surface area, which contributes to its consistent reactivity under the given solvent conditions. Mon_MolWt yields + 3.15 pps, confirming that DMA, which is the lightest monomer with a molecular weight of 99 g·mol^−1^, operates well within the kinetically controlled regime where diffusion does not limit reaction rates. RAFT agent contributions are uniformly positive but smaller in magnitude: RAFT_SMR_VSA6 (+ 1.87 pps) indicates good polarizability matching with DMA, RAFT_EState_VSA7 (+ 1.52 pps) suggests optimal electronic complementarity, and RAFT_VSA_EState5 (+ 1.31 pps) confirms favorable electronic surface interactions. These positive contributions, although individually modest, collectively facilitate chain transfer and improve monomer conversion. The 76 additional features contribute + 24.01 pps, demonstrating that many minor favorable interactions accumulate to create ideal polymerization conditions. This sample indeed matches the DMA-BTPA combination, supporting the mechanistic hypothesis from the waterfall plot: the harmonization of minimal steric barriers, optimal electronic properties, effective mass transport, and high RAFT agent compatibility provides ideal conditions for PET-RAFT polymerization.

The opposing waterfall plots illustrate different mechanistic scenarios: the sample with low conversion value faces cumulative disadvantages, including high topological complexity, polarized electronics, high molecular weight, and poor RAFT matching, creating a challenging environment. Conversely, the sample exhibiting high conversion benefits from a combination of advantages arising from its simple structure, radical delocalization, low molecular weight, and well-matched RAFT agent, which facilitate efficient polymerization. The approximately 49-pps deviation from the average monomer conversion indicates PET-RAFT’s extreme sensitivity to molecular design choices. In our specific case, monomer descriptors are the most influential, followed by RAFT descriptors, in both plots. These local insights support our global SHAP analysis: Mon_Kappa3 exhibits the most significant individual contributions, while Mon_MaxEStateIndex and Mon_MolWt consistently rank among the top contributors, with effects that align with mechanistic expectations. This harmony between global importance and local contributions indicates that our model has captured meaningful chemical relationships rather than random correlations, reinforcing confidence in its predictive power and mechanistic explanations for rational PET-RAFT design.

### External validation and generalizability assessment

To assess the CatBoost’s ability to perform beyond the internal test set, we conducted divergent external validation with nine independent PET-RAFT polymerization experiments that studied different monomer-RAFT agent combinations^38^. Table 1 presents the generalization performance of CatBoost on the respective monomers polymerized under identical reaction conditions. To evaluate the generalizability of CatBoost, we selected structurally diverse monomers, including MA, DEA, and NAM, while maintaining the RAFT agent and photocatalyst types consistent with those of the training set. By doing so, we assessed the model’s ability to transfer learned QSPPRs to unknown PET-RAFT systems beyond the training domain.

**Table 1:** External validation results demonstrate the CatBoost model’s generalization performance across varying combinations of components in unknown PET-RAFT systems.

Monomer^†^	RAFT agent	Photocatalyst	Experimental monomer conversion (%)	Predicted monomer conversion (%)	Absolute error (pps)	Error metrics (pps)
MA	R1	ZnTPP	98.00	87.75	10.25	RMSE = 8.16 MAE = 6.47
DMA	R1		97.00	96.17	0.83	
DEA	R1		99.00	87.15	11.85	
NAM	R1		97.00	87.69	9.31	
BzMA	R2		91.00	90.89	0.11	

^†^Monomers include MA: methyl acrylate, DMA: N, N-dimethylacrylamide; DEA: N, N-diethylacrylamide; NAM: N-acryloylmorpholine; BzMA: benzyl methacrylate. RAFT agents include BTPA: 2-(butylthiocarbonothioylthio) propionic acid (R1); CDTPA: 4-cyano-4-[(dodecylsulfanylthiocarbonyl)sulfanyl]pentanoic acid (R2). The photocatalyst is ZnTPP: zinc(II) tetraphenylporphyrin. “Experimental” and “Predicted” monomer conversion (%)” correspond to the actual monomer conversions obtained from literature sources and CatBoost model predictions, respectively. Absolute errors are computed as the absolute difference between the experimental and predicted conversion values and are expressed in percentage points (pps). External validation dataset conditions: PET-RAFT reactions were performed under yellow LED light (560 nm, 9.7 mW cm^−2^) using a [M]:[RAFT]:[ZnTPP] = 50:1:0.02 and with [M_0_] = 1 M in a 4-h reaction time. All experiments were conducted under ambient conditions in 96-well plates, with 200 µL of reaction mixture per well^38^.

Table 1 summarizes the external validation test results, evaluating the generalizability of the CatBoost regressor. Despite having no prior information about the altered component combinations, the model maintains a reasonable predictive performance through its learned understanding of QSPPR patterns. Validation errors for the external set ranged from 0.11 to 11.85 pps, with 60% of predictions falling within the training MAE (9.62 ± 1.71 pps) and 100% within the training MAE ± 2SD band, despite testing different monomers (DEA, NAM, MA) than those of the training set. This performance is particularly captivating given that the model cannot account for wavelength shifts, intensity changes, or stoichiometric variations. Yet, it relies entirely on the intrinsic chemical properties encoded in the molecular descriptors, underscoring the importance of molecular descriptors and chemical similarity between reacting components in monomer conversion prediction, rather than on experimental conditions.

CatBoost achieved a significantly high validation performance (RMSE = 8.16 pps and MAE = 6.47 pps) across both acrylate and acrylamide families, outperforming its training performance (RMSE = 12.18 ± 1.81 pps, MAE = 9.62 ± 1.71 pps). Among various monomers, MA showed the lowest absolute error of 10.25 pps. BzMA showed an exceptionally low error (0.11 pps), confirming that Mon_Kappa3, our primary SHAP predictor (see Sect. “Mon_Kappa3”), effectively captures steric effects regardless of stoichiometric variations. DMA had a very low error (0.83 pps), and previously unseen NAM and DEA showed acceptable errors (9.31 and 11.85 pps, respectively) in monomer-conversion prediction. These results indicate that electronic descriptors (VSA_EState, MaxEStateIndex) effectively capture the delocalized amide resonance, thereby creating a similar electronic environment across acrylamide monomers. DEA’s longer alkyl substituents, however, likely conferred greater conformational flexibility that 2D descriptors may not fully capture, which explains its slightly higher error relative to DMA and NAM. The exceptional performance of BzMA (absolute error of 0.11 pps) indicates that our topological descriptors, particularly the Mon_Kappa3 shape index, which emerged as the most critical feature in SHAP analysis, effectively capture the reactivity patterns of bulky monomers.

Overall, although the model cannot explicitly account for parameter variations, its ability to maintain reasonable predictive accuracy via learned QSPPR patterns supports our focus on intrinsic molecular features rather than experimental parameters. The successful external validation of CatBoost across diverse monomers confirms that our model has captured fundamental chemical principles rather than merely memorizing the training data.

## Conclusion and future work

This study, to our knowledge, demonstrates the first successful application of molecular descriptor-based ML for monomer conversion prediction in PET-RAFT polymerization. By breaking down the PET-RAFT system into its molecular components (monomer, RAFT agent, and photocatalyst), encoding each component with SMILES-derived descriptors in addition to thermodynamic features (i.e., the radical stabilization energy, the fragmentation efficiency, and enthalpy values of RAFT stability) for the RAFT agents, we trained ten different ML algorithms. Given the relatively small dataset size (n = 152) used to train the ML models, several precautions were taken to prevent overfitting. First, we benchmarked multiple ML models and prioritized ensemble methods (i.e., RF/GB/CatBoost), which inherently incorporate regularization mechanisms, such as bootstrap aggregation, learning-rate shrinkage, and depth limits, and are particularly well-suited for small, descriptor-rich datasets^61^. Second, the dimensionality of the initial design space was reduced from 217 to 83 features via variance- and correlation-based filtering, thereby reducing noise and multicollinearity^62^. Third, and most importantly, a fivefold CV followed by stability assessment within each fold (SD of R^2^ = ± 0.07 for the CatBoost regressor) ensured maximal use of the available data during model training and enabled selection of the most robust model across data partitions ^20, 70^. Among 10 ML models, the CatBoost regressor was identified as the top performer because it exhibited the highest training stability across CV folds (SD = ± 0.07) and achieved the best prediction performance for the test set (R^2^ = 0.84; RMSE = 10.04 pps; MAE = 8.16 pps). Next, CatBoost predictions were interpreted using SHAP analyses, which provided both global and local insights into how molecular descriptors influenced individual predictions. Unlike black-box ML models and chemoinformatic tools that rely on one-hot-encoded polymer-forming components and/or experimental variables for QSPPR prediction, the explainable ML model presented here can interpret the physicochemical factors that influence monomer conversion in a specific PET-RAFT system. External validation across diverse PET-RAFT component combinations demonstrated the model’s strong generalizability to monomers beyond the training set. These include MA, DEA, and NAM, which are part of the broader (meth)acrylate and (meth)acrylamide families but differ in size, polarity, and substitution patterns. The validation results showed an MAE of 6.47 pps, comparable to the prediction error during CatBoost’s training (MAE = 9.62 ± 1.71 pps). A larger error of 11.85 pps was observed with the DEA-based PET-RAFT system, likely because longer alkyl substituents confer greater conformational flexibility that 2D descriptors do not fully account for.

Since this study is the first to fully implement a molecular descriptor-based ML approach for PET-RAFT monomer conversion prediction, there is still significant potential for model improvement. First, relying on 2D molecular descriptors inherently cannot capture conformational dynamics, photophysical properties of photocatalysts (i.e., quantum yields), π-π stacking interactions, or solvent-specific effects that influence polymerization kinetics^63^. Incorporating 3D descriptors or features derived from molecular dynamics simulations could address these limitations, albeit at the cost of the resource-intensive nature of such computational approaches. Second, training the model exclusively on molecular descriptors prevents it from accounting for variations in reaction processes or stoichiometry, even though the validation studies showed that this limitation affected only specific polymers (DEA and NAM) in the dataset. The model generalizes well by capturing intrinsic molecular properties; however, the same feature prevents it from accounting for reaction processes or stoichiometric variations. Yet, these limitations paradoxically validate our approach: the model’s reasonable prediction accuracy (R^2^ = 0.84) despite these constraints highlights the importance of molecular descriptors for predicting monomer conversion.

Future improvements should address these limitations while maintaining interpretability. For example, combining explainable ML with high-throughput experimentation platforms could enable exploration of a more expansive chemical space and expand the training data, thereby enhancing prediction accuracy. Adding physics-informed constraints would improve generalization performance. Furthermore, the wider literature offers promising avenues for implementation that, when combined with our approach, are likely to enable mechanism-aware, closed-loop polymer design. These include (i) inverse design with reinforcement learning to generate component sets meeting target objectives^64^, (ii) active-learning architectures that couple kinetic Monte Carlo with query-efficient selection to learn molecular weight distribution and kinetics with few simulations/experiments^65^, and (iii) models that infer kinetic parameters (e.g., ${k}_{p}$) from monomer structure, providing physics-aware priors for hybrid surrogates^66^. Combining these approaches effectively with the presented explainable techniques and integrating them into closed-loop autonomous technologies can shorten the design-build-test-learn cycles while still enabling researchers to uncover QSPPR relationships. This is precisely what is needed to scale PET-RAFT toward autonomous, Industry 4.0-aligned synthesis, with the added benefit of explainability.

In conclusion, this work demonstrates that accepting slightly higher prediction uncertainty by excluding experimental parameters and using solely molecular descriptors yields invaluable mechanistic transparency. As polymer science increasingly adopts autonomous synthesis and AI-driven discovery, explainable models that uncover QSPPRs will become essential bridges between machine intelligence and mechanistic insights, potentially guiding researchers in designing novel component formulations^10^. The success achieved with only 152 PET-RAFT sets demonstrates that integrating molecular descriptor-based ML with expert domain knowledge can mitigate data scarcity, providing a roadmap for developing diverse ML models for specialized polymerization systems where large datasets remain unavailable. While this study demonstrates the prediction of monomer-to-polymer conversion under fixed experimental conditions, it also serves as an essential preliminary step toward predicting molecular weight distribution. Ongoing efforts aim to expand the explainable ML approach presented herein to photoiniferter (PI)-RAFT polymerization for AI-guided macromolecular design.

## Materials and methods

### Data collection and curation

A high-throughput PET-RAFT dataset from the Boyer team was used in this work for ML training and yield-prediction performance evaluation^30^. In summary, the dataset encompasses four different monomers: *N*, *N*-dimethylacrylamide (DMA, 99%), benzyl acrylate (BzA), benzyl methacrylate (BzMA, 97%) and 2-hydroxypropyl methacrylamide (HPMA, 99%), which are polymerized under ambient conditions in 96-well plates with a 200 µL volume. Polymerization reactions were conducted in dimethyl sulfoxide (DMSO) using a monomer concentration of 1.0 M, with a fixed monomer:RAFT agent:photocatalyst molar ratio ([M]:[R]:[PC]) of 100:1:0.015. The reactions were initiated by irradiation with green LED light at 530 nm and 5.5 mW cm^−2^ for 4 or 24 h (for HMPA). The study screened ten RAFT agents, including trithiocarbonates, dithiocarbamates, dithioesters, and xanthates, along with four zinc-based metalloporphyrin photocatalysts. Monomer conversion or polymerization yield (%) was measured by proton nuclear magnetic resonance (^1^H NMR) spectroscopy and was designated as the target feature in subsequent ML models to predict. To visualize the distribution of monomer conversion data, please refer to the *Supporting Document* (Figure S1).

### Molecular descriptor calculation

All chemical structures that form the building blocks of the PET-RAFT polymerization reaction (monomers, RAFT agents, and photocatalysts) were represented as SMILES strings and processed with RDKit (v2023.09.1) to compute a comprehensive set of 2D molecular descriptors. The presented approach differs fundamentally from traditional chemoinformatics, as it separately encodes the molecular descriptors of each reaction component rather than treating the system as a single, aggregated entity.

For each component, we calculated 208 descriptors, including surface area, electronic and charge descriptors, as well as topological and shape indices. Additionally, we included three key RAFT agent-specific thermodynamic properties from the literature: the radical stabilization energy, the fragmentation efficiency, and the enthalpy of RAFT stability ^30, 67^

Data preprocessing was performed to reduce variance and multicollinearity through variance filtering (threshold < 0.05) and correlation analysis (|r|> 0.95)^68, 69^. After feature selection and dimensionality reduction, we retained 83 features in total for the RAFT agent (prefixed “RAFT_”), monomer (“Mon_”), and photocatalyst (“Photo_”), respectively. Ultimately, feature scaling was performed using z-score normalization, in which all continuous features were subtracted from their mean and scaled to unit variance, ensuring that features maintain the same scale and preventing any one feature from dominating others, thereby mitigating bias^76, 70^. The complete descriptor list and thermodynamic properties are detailed in Table S2**.**

### Model development, stability evaluation, and performance testing

We implemented ML models using scikit-learn (v1.x) and specialized libraries for XGBoost v3.0.4, CatBoost v1.2.8. Model development was performed in Python using the Google Colab environment^71^, with GPU acceleration enabled for neural network training. We systematically trained and evaluated 10 ML models, encompassing (i) linear models, including LR, Ridge, Lasso; (ii) kernel method: SVR; (iii) an instance-based learner: k-NN; (iv) ensemble tree-based methods such as RF, GBR, XGBoost, and CatBoost; as well as a neural network: MLP. The complete algorithm specifications are summarized in Table S3**.**

Performance evaluation employed three complementary metrics throughout training and testing, with mathematical definitions provided below, where ${y}_{i}$ represents actual values, $\widehat{{y}_{i}}$ represents predicted values, $\overline{y}$ represents the mean of actual values, and $n$ represents the number of data points.

The coefficient of determination, R^2^ (**Eq. **1), is a dimensionless metric that indicates the proportion of variance in the dependent variable explained by the model’s independent variables. It ranges from -$\infty$ to 1, with values closer to 1 indicating better model performance. It is used as the primary metric to evaluate model accuracy, measuring the proportion of variance in monomer conversion explained by molecular descriptors.

1 $${R}^{2}=1-\frac{{\sum }_{i=1}^{n}{\left({y}_{i}-\widehat{{y}_{i}}\right)}^{2}}{{\sum }_{i=1}^{n}{\left({y}_{i}-\overline{y}\right)}^{2}}$$

The Root Mean Squared Error (RMSE), as given in **Eq. **2**,** measures the average magnitude of prediction errors, with greater sensitivity to large errors due to the squaring process. In our PET-RAFT dataset, RMSE values of 12.18% suggest that predictions typically deviate from actual conversion values by approximately 12 pps, with greater weight given to larger prediction errors.

2 $$RMSE=\sqrt{\frac{1}{n}{\sum }_{i=1}^{n}{\left({y}_{i}-\widehat{{y}_{i}}\right)}^{2}}$$

The Mean Absolute Error (MAE) measures the average absolute difference between predicted and actual values (**Eq. **3). It is less sensitive to outliers than RMSE and provides an intuitive measure of typical prediction error. The MAE indicates the average absolute difference between the predicted and actual conversion percentages, providing a straightforward way to understand typical prediction accuracy by treating all errors equally, regardless of their magnitude.

3 $$MAE=\frac{1}{n}{\sum }_{i=1}^{n}\left|{y}_{i}-\widehat{{y}_{i}}\right|$$

Following feature selection and data preprocessing, we split the PET-RAFT dataset into an 80/20 split: 80% of the dataset was allocated as a training set, used for hyperparameter tuning and model training via *k*-fold CV (*k* = 5). Model training employed a *k*-fold CV approach, which randomly splits the training set into *k* equal subsets, using *k*−1 parts for training and one part for validation in each round. It repeats this process *k* times so that each part serves as validation data once. This approach provides a reliable performance estimate by averaging results across folds, thereby reducing variance while maximizing the utilization of available training data, which is especially beneficial for small datasets^20^. The parity plots visualize the predicted versus actual monomer conversion for both model training and testing (Fig. 4a-h**)**. The detailed analyses for training performance comparison include R^2^, RMSE, and MAE. Model stability was assessed by examining the ± SD of R^2^ values across the CV folds^68, 69^. Although atypical in standard ML studies, we further provided statistical evidence for model stability by applying a paired *t*-test to compare the mean R^2^ values obtained for CatBoost with those of each competing model (**p < 0.01, **p < 0.05;* p* > 0.05, *n.s*. = not significant).

Following model training and stability testing, the held-out test set (20% of the initial data, n ≈ 30) was used to assess the model’s ability to predict monomer conversion. This testing phase provides an unbiased estimate of prediction performance, as the test set was never exposed to any model during training, hyperparameter optimization, or CV steps. Parity plots comparing predicted versus actual conversion values on the test set (Figs. 4b, 4 d, 4f, and 4 h) provide a visual assessment of the model’s predictive performance, with ideal predictions indicated by tight clustering along the diagonal line (x = y). The optimal model was selected based on model stability during training and accuracy, and was subsequently used to predict monomer conversion.

### External validation

External validation is critical for demonstrating the practical applicability and generalizability of an ML model beyond its training domain. The validation set included MA, DMA, DEA, NAM, and BzMA: DMA and BzMA were present in the training set, while MA, DEA, and NAM represented previously unseen molecular structures (Fig. 3b). The reactions utilized BTPA or CDTPA as RAFT agents with ZnTPP photocatalyst. Some of the combinations were present in the training data but under different experimental conditions: the wavelength was shifted from 530 to 560 nm, the light intensity increased from 5.5 to 9.7 mW cm^−2^, and the monomer-to-RAFT ratio was varied from the training standard of 100:1 to 50:1.

Validation performance was assessed by computing MAE and RMSE on the external set and comparing them with the fivefold CV-trained CatBoost performance **(**Table 1**).**

### Evaluation of model explainability

SHAP (SHapley Additive exPlanations) analyses enable interpreting the predictions made by an ML model using principles from game theory^22, 72^. It assigns each feature a fair Shapley value (${\Phi }_{i}$), reflecting its unique contribution to the model’s output relative to other features, by averaging the marginal contribution of each feature across all subsets of descriptors. The equation used to calculate the SHAP value for a single feature $i$ is given below (**Eq. **4)^73^. In this equation, ${\Phi }_{i}$ denotes the unique contribution (SHAP value) of feature $i$; $N$ is the complete set of $M$ features; $S$ is any subset of features not containing feature $i$ (with size $\left|S\right|$); $f$(⋅) is the model output so f $\left[f\left(S\cup \{i\}\right)-f\left(S\right)\right]$ is the marginal effect of adding $i$ to $S$; and the factorial weight $\frac{\left|S\right|!\hspace{0.17em}\left(M-\left|S\right|-1\right)!}{M!}$ equals the fraction of all $M!$ feature orderings in which precisely the features in $S$ appear before $i$. These SHAP values are additive: their total sum equals the difference between the model’s prediction and the average prediction. A positive SHAP value indicates that the feature raises the prediction above average, while a negative value lowers it. By examining SHAP value distributions or averages, one gets global interpretations (ranking feature impacts) or local ones (direction of each feature on a single prediction).

4 $${\Phi }_{i}={\sum }_{S\subseteq N\setminus \{i\}}\frac{\left|S\right|!\hspace{0.17em}\left(M-\left|S\right|-1\right)!}{M!}\left[f\left(S\cup \{i\}\right)-f\left(S\right)\right],\hspace{1em}i\in N$$

To ensure mechanistic transparency and extract chemically meaningful insights from the top-performing model, CatBoost, we conducted a comprehensive interpretability assessment by SHAP analyses. For global insight, we aggregated SHAP values across the dataset by computing the mean absolute SHAP value for each descriptor and ranking the features by their relative impact on predicting the conversion percentage using beeswarm and bar charts (Figs. 5** and S5**). To complement the global feature importance analysis, we generated SHAP waterfall plots and examined individual prediction mechanisms for specific samples at the extremes of our conversion distribution (Figs. 6** and S6**).

Waterfall plots provide local interpretability by decomposing a single prediction into additive feature contributions, starting from the baseline, $E\left[\mathrm{f}\left(\mathrm{x}\right)\right]$, (the mean prediction across the training set), and sequentially adding each feature’s SHAP value to reach the final model prediction^74^. This additive decomposition satisfies the local accuracy property, meaning that the sum of all SHAP values plus the baseline equals the model’s output. $\phi_i$ denotes the SHAP value for feature *i* (**Eq. **5).

5 $$[f(x)] = E[f(x)] + \sum {\varphi_{i} }$$

## Supplementary Information

Supplementary Information.

## Data Availability

The curated dataset and the Python code used for model training and analysis are available from the corresponding author upon reasonable request.
