# Supplementary material for: Leveraging molecular descriptors and explainable machine learning for monomer conversion prediction in photoinduced electron transfer-reversible addition-fragmentation chain transfer polymerization
Source: Sci Rep. 2026 Feb 9;16:5947. doi: 10.1038/s41598-025-33553-y (PMC12894868; doi:10.1038/s41598-025-33553-y)
Supplement: Supplementary file 1 — Supplementary Information. [file 41598_2025_33553_MOESM1_ESM.pdf]

SUPPORTING DOCUMENT

Leveraging Molecular Descriptors and Explainable Machine Learning for Monomer Conversion Prediction in Photoinduced Electron Transfer-Reversible Addition-Fragmentation Chain Transfer Polymerization

Berna Alemdag[a], Azra Kocaarslan[a,b,*], Gözde Kabay[a,*]

[a] Karlsruhe Institute of Technology (KIT), Institute of Functional Interfaces (IFG), Department for Bioengineering and Biosystems, 76344 Eggenstein-Leopoldshafen, Germany.
[b] Karlsruhe Institute of Technology (KIT), Institute of Nanotechnology (INT), Department for Bioengineering and Biosystems, 76344 Eggenstein-Leopoldshafen, Germany.

E-mail: *gozde.kabay@kit.edu*; *azra.kocaarslan@kit.edu*

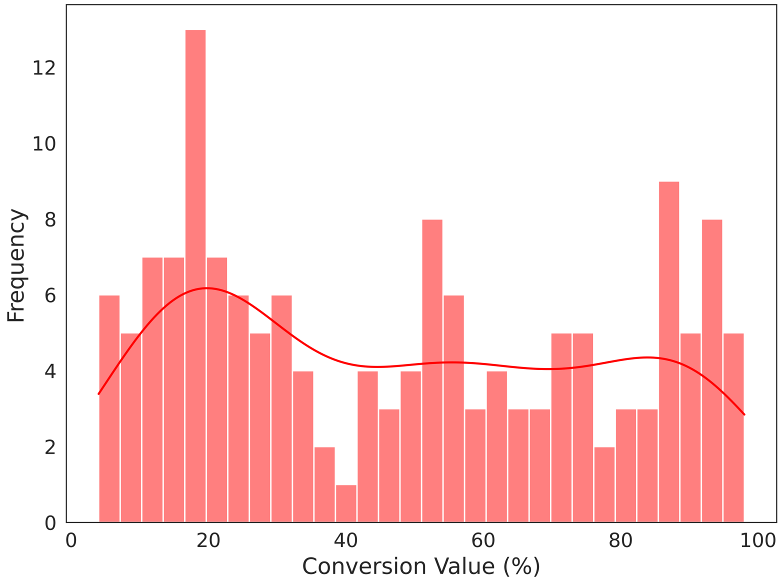

Figure S1. This histogram illustrates the distribution of monomer conversion values expressed as a percentage (%) across the sampled data. The x-axis represents the conversion value ranging from 0% to 100%, while the y-axis indicates the frequency of occurrences for each conversion range. A red trend line overlays the histogram, indicating the general trend of the data distribution.

Table S1. Summary of the monomer conversion data distribution obtained from 152 PET-RAFT sets used as target variables for model development and testing.

**Parameter**	**Value**
Standard deviation	29.27
Minimum value (%)	4.00
25th percentile (%)	19.75
50th percentile (%)	47.5
75th percentile (%)	74.25
Maximum value (%)	98.00
Number of features	83

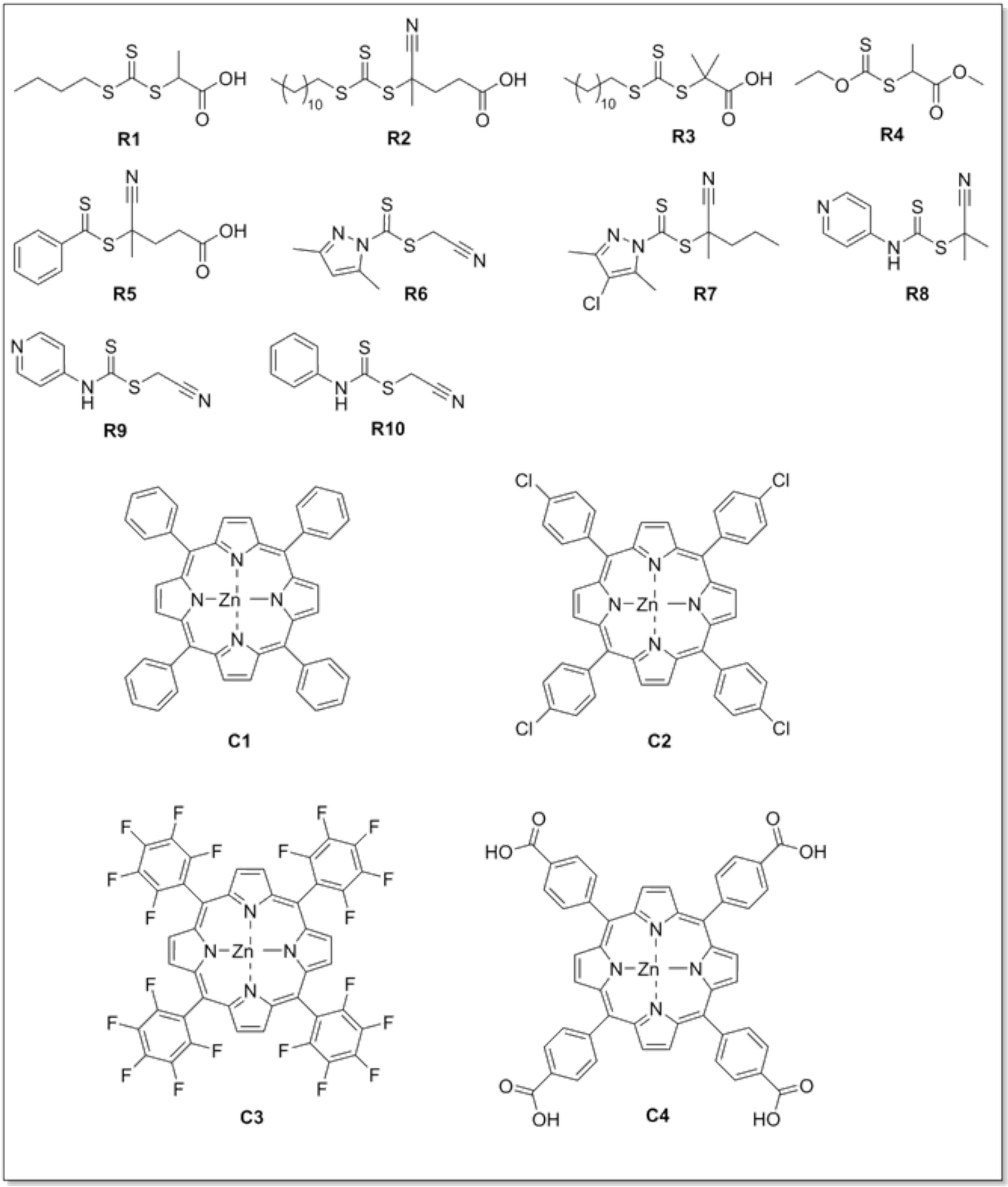

Figure S2. Chemical structures of the RAFT agents and photocatalysts. R1–R10 demonstrate RAFT agents. C1–C4 demonstrate zinc porphyrin-based photocatalysts with different meso-substituents: C1 (phenyl), C2 (dichlorophenyl), C3 (pentafluorophenyl), and C4 (carboxyphenyl).

Table S2. A list of RDKit descriptors and thermodynamic properties utilized for model training in this study.[1]

Category	Input Features
	Molecular Descriptors from RDKit
Hydrophobicity/surface area descriptors	SlogP_VSA1, SlogP_VSA2, SlogP_VSA3, SlogP_VSA4, SlogP_VSA5, SlogP_VSA6, SlogP_VSA7, SlogP_VSA8, SlogP_VSA9, SlogP_VSA10, SlogP_VSA11, SlogP_VSA12
Polarizability/dispersion interaction descriptors	SMR_VSA1, SMR_VSA2, SMR_VSA3, SMR_VSA4, SMR_VSA5, SMR_VSA6, SMR_VSA7, SMR_VSA8, SMR_VSA9, SMR_VSA10
Topology and electronic distribution descriptors	VSA_EState1, VSA_EState2, VSA_EState3, VSA_EState4, VSA_EState5, VSA_EState6, VSA_EState7, VSA_EState8, VSA_EState9, VSA_EState10
Electrostatic / charge descriptors	PEOE_VSA1, PEOE_VSA2, PEOE_VSA3, PEOE_VSA4, PEOE_VSA5, PEOE_VSA6, PEOE_VSA7, PEOE_VSA8, PEOE_VSA9, PEOE_VSA10, PEOE_VSA11, PEOE_VSA12, PEOE_VSA13, PEOE_VSA14
Electronic state / topological descriptors	EState_VSA1, EState_VSA2, EState_VSA3, EState_VSA4, EState_VSA5, EState_VSA6, EState_VSA7, EState_VSA8, EState_VSA9, EState_VSA10, EState_VSA11
Global polarity / H-bonding capacity descriptor	TPSA
Global size/shape descriptor	LabuteASA
Molecular weight descriptor	MolWt, HeavyAtomMolWt, ExactMolWt
Electronic descriptors	NumValenceElectrons, NumRadicalElectrons
Charge descriptors	MaxPartialCharge, MinPartialCharge, MaxAbsPartialCharge, MinAbsPartialCharge
Morgan fingerprint density descriptors	FpDensityMorgan1, FpDensityMorgan2, FpDensityMorgan3
Shape indices	Kappa1, Kappa2, Kappa3
Topological descriptors	RingCount, BalabanJ, BertzCT
EState indices	MaxAbsEStateIndex, MinAbsEStateIndex, MaxEStateIndex, MinEStateIndex
Chi descriptors	Chi0, Chi0n, Chi0v, Chi1, Chi1n, Chi1v, Chi2n, Chi2v, Chi3n, Chi3v, Chi4n, Chi4v
Molar logarithm of the partition coefficient (logP)	MolLogP
The fraction of sp3-hybridized carbon atoms in a molecule.	FractionCSP3
Alpha value of Hall-Kier	HallKierAlpha
Coefficient of the characteristic polynomial of the adjacency matrix of the suppressed molecular graph	Ipc
Specific functional group present	fr_Al_COO, fr_Al_OH, fr_Al_OH_noTert, fr_ArN, fr_Ar_COO, fr_Ar_N, fr_Ar_NH, fr_Ar_OH, fr_COO, fr_COO2, fr_C_O, fr_C_O_noCOO, fr_C_S, fr_HOCCN, fr_Imine, fr_NH0, fr_NH1, fr_NH2, fr_N_O,fr_Ndealkylation1, fr_Ndealkylation2, fr_Nhpyrrole, fr_SH, fr_aldehyde, fr_alkyl_carbamate, fr_alkyl_halide, fr_allylic_oxid, fr_amide, fr_amidine, fr_aniline, fr_aryl_methyl, fr_azide, fr_azo, fr_barbitur, fr_benzene, fr_benzodiazepine, fr_bicyclic, fr_diazo, fr_dihydropyridine, fr_epoxide, fr_ester, fr_ether, fr_furan, fr_guanido, fr_halogen, fr_hdrzine, fr_hdrzone, fr_imidazole, fr_imide, fr_isocyan, fr_isothiocyan, fr_ketone, fr_ketone_Topliss, fr_lactam,fr_lactone, fr_methoxy, fr_morpholine, fr_nitrile, fr_nitro, fr_nitro_arom, fr_nitro_arom_nonortho,

	fr_nitroso, fr_oxazole, fr_oxime, fr_para_hydroxylation, fr_phenol, fr_phenol_noOrthoHbond, fr_phos_acid, fr_phos_ester, fr_piperdine, fr_piperzine, fr_priamide, fr_prisulfonamd, fr_pyridine, fr_quatN, fr_sulfide, fr_sulfonamd, fr_sulfone, fr_term_acetylene, fr_tetrazole, fr_thiazole, fr_thiocyan, fr_thiophene, fr_unbrch_alkane, fr_urea.

Thermodynamic properties of RAFT agents[2–4]

RAFT agent type	ΔH_{stab} (kJ mol^{-1})	ΔH_{frag} (kJ mol^{-1})	RSE (kJ mol^{-1})
BTPA	59.4	-4.4	41.3
CDTPA	59.4	-4.4	59.0
DDMAT	59.4	-4.4	54.9
Xanthate	86.8	-44.3	41.3
CPADB	41.3	+21.0	59.0
DTC1	47.9	-5.5	31.9
DTC2	45.0	-3.2	59.0
CBPCD	90.6	-32.7	59.0
CMPCD	90.6	-32.7	31.9
CMPhCD	90.6	-32.7	31.9

*Enthalpy values of RAFT stability (ΔH_{stab}), the fragmentation efficiency (ΔH_{frag}), and the radical stabilization energy (RSE) of the leaving R$^{\bullet}$ group radicals (kJ/mol) for the various RAFT agents. List of RAFT agents employed in this work composed of trithiocarbonates (including, 2-(butylthiocarbonothioylthio) propanoic acid (BTPA, R1), 4-cyano-4-[(dodecylsulfanylthiocarbonyl)sulfanyl]pentanoic acid (CDTPA, R2), 2-(dodecylthiocarbonothioylthio)-2-methylpropionic acid (DDMAT, R3), methyl 2-((ethoxycarbonothioyl)thio)propanoate (Xanthate, R4), 4-cyano-4-(phenylcarbonothioylthio)pentanoic acid (CPADB, R5), cyanomethyl (3,5-dimethyl-1H-pyrazole)-carbodithioate (DTC1, R6), and 2-cyanobutan-2-yl 4-chloro-3,5-dimethyl-1H-pyrazole-1-carbodithioate (DTC2, R7), 2-cyanobutan-2-yl methyl(pyridin-4-yl)carbamodithioate (CBPCD, R8), cyanomethyl methyl(4-pyridyl)carbamodithioate (CMPCD, R9), and cyanomethyl methyl(phenyl)carbamodithioate (CMPhCD, R10).

Table S3. Summary of the ML models evaluated in this study. The table outlines the model types, key hyperparameters, and essential features.

Model	Type	Key parameters	Nonlinearity
LR	Linear	None (baseline)	No
Ridge	Linear (L2)	alpha=1.0	No
Lasso	Linear (L1)	alpha=0.1	No
SVR	Kernel	C=1.0<br>kernel= "rbf"	Yes<br>(via kernel)
k-NN	Instance-based	k=5	Implicit<br>(local)
RF	Ensemble (Bagging)	n_estimators=100<br>random_state=42<br>max_depth=None	Yes
GBR	Ensemble (Boosting)	random_state=42<br>n_estimators=100	Yes
XGBoost		n_estimators=100<br>random_state=42<br>verbosity=0	Yes
CatBoost		early_stopping=True<br>verbose=0<br>random_state=42	Yes
MLP	Artificial Neural Network	hidden_layer_sizes = (128, 64)<br>max_iter =300	Yes

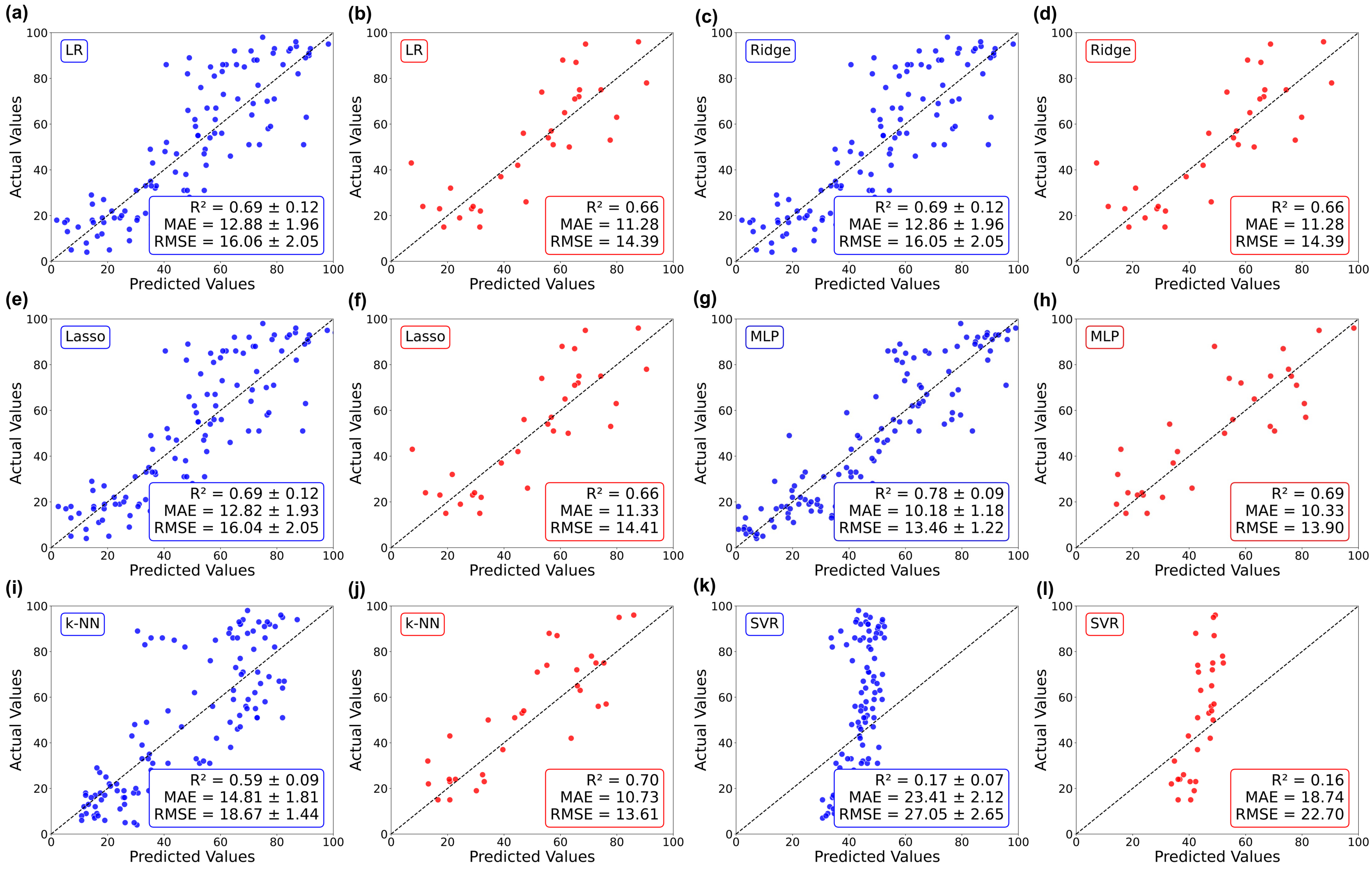

Figure S3. Parity plots demonstrating predicted versus actual monomer conversion (%) values for (a, b) Linear Regression (LR), (c, d) Ridge, (e, f) Lasso, (g, h) Multi-Layer Perceptron (MLP), (i, j) k-Nearest Neighbors (k-NN), and (k) Support Vector Regression (SVR). The blue-dotted graphs correspond to predictions obtained during 5-fold CV on the training set, while the red-dotted plots show predictions on the held-out test. RMSE and MAE values represent data in pps. The dashed diagonal line (x = y) indicates perfect prediction, where the predicted and actual values match.

Table S4. A summary of model training performance metrics with *5*-fold CV.

ML model	R^2	R^2_SD	RMSE (pps)	RMSE_SD	MAE (pps)	MAE_SD
XGBoost	0.83	0.11	11.51	3.33	8.14	2.18
CatBoost	0.82	0.07	12.18	1.81	9.62	1.71
RF	0.82	0.10	11.88	2.60	9.25	2.39
GBR	0.81	0.09	12.54	2.27	9.06	1.83
k-NN	0.59	0.09	18.67	1.44	14.80	1.81
MLP	0.78	0.09	13.46	1.22	10.18	1.18
LR	0.69	0.12	16.06	2.04	12.88	1.96
Ridge	0.69	0.12	16.05	2.05	12.86	1.96
Lasso	0.69	0.12	16.04	2.05	12.82	1.93
SVR	0.17	0.07	27.05	2.65	23.41	2.12

Table S5. Comparison of model test performance metrics for monomer conversion prediction on the held-out test set.

**ML model**	**R^2**	**RMSE**	**MAE**
CatBoost	0.84	10.04	8.16
RF	0.84	9.93	8.17
GBR	0.78	11.59	8.98
XGBoost	0.77	11.80	9.25
k-NN	0.70	13.61	10.73
MLP	0.69	13.90	10.33
LR	0.66	14.39	11.28
Ridge	0.66	14.39	11.28
Lasso	0.66	14.41	11.31
SVR	0.16	22.70	18.74

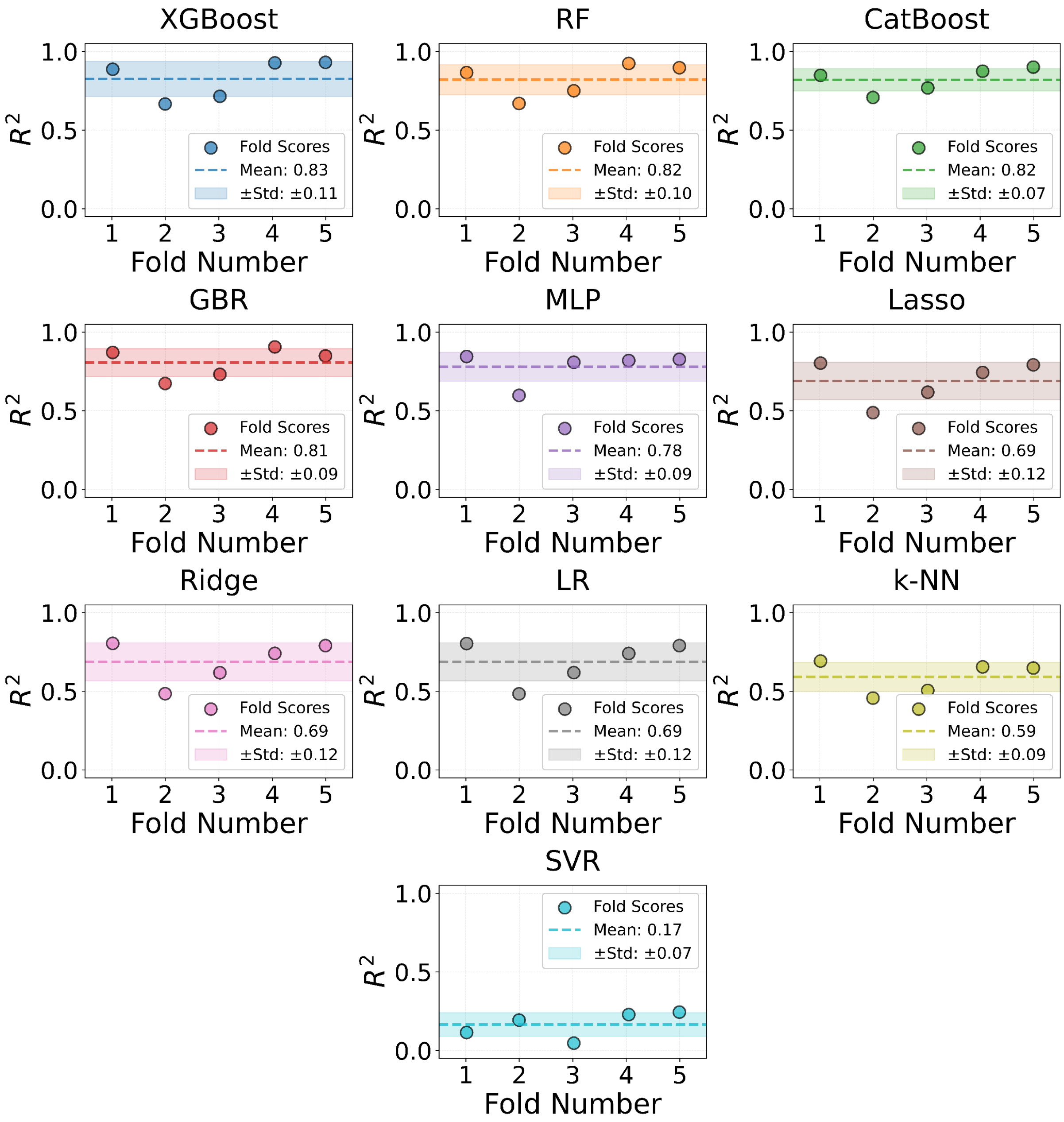

Figure S4. The stability and performance of 10 ML models are presented using 5-fold cross-validation. Each subplot displays individual fold R^2 scores as points, with dashed lines indicating the mean R^2 values. The shaded area represents the standard deviation (±SD), with a smaller SD indicating greater model stability across data splits.

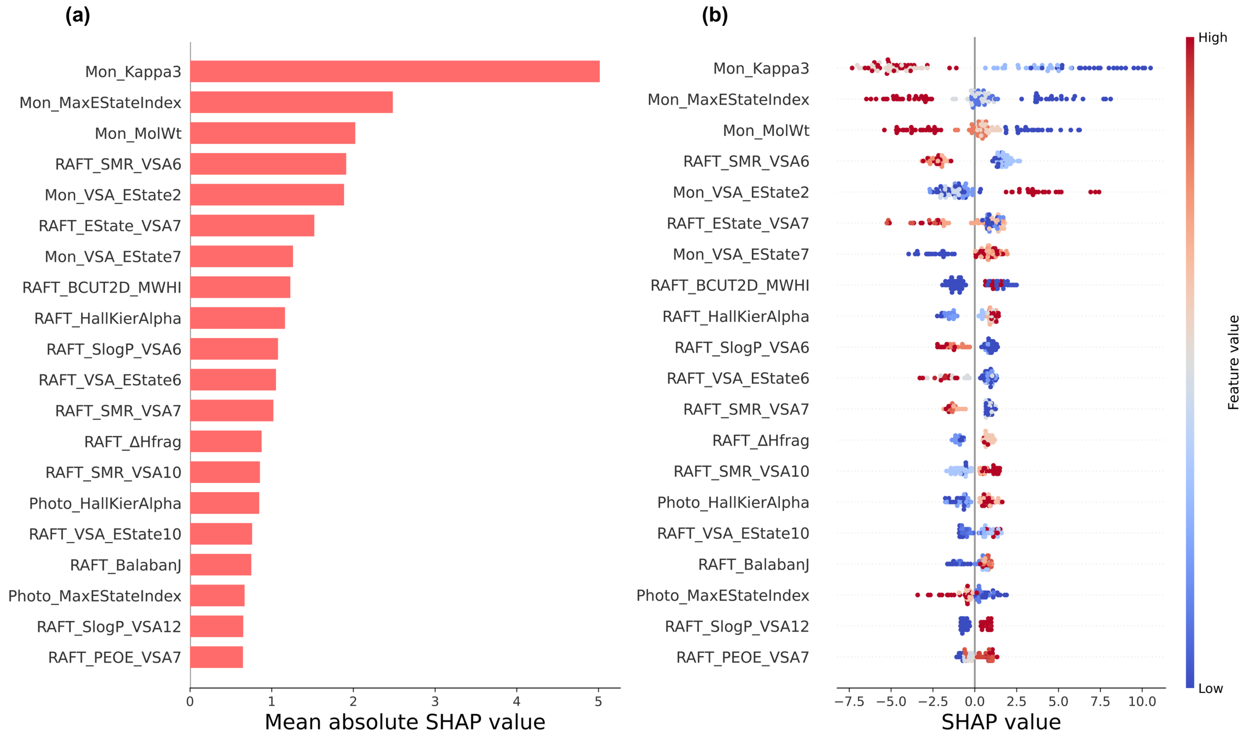

Figure S5. Global feature importance ranking for the CatBoost model. (a) The bar chart displays the mean absolute SHAP values for input parameters, indicating their overall contribution to the model output. Higher mean absolute SHAP values (red bars) indicate greater predictive impact of the individual feature across the dataset. (b) The beeswarm plot illustrates the spread of the effects of each molecular descriptor on conversion prediction across 100 randomly selected samples from the dataset. Each point corresponds to a single sample. Blue (low values) indicates that a feature has a negative influence on the predicted outcome, whereas red (high values) indicates a positive impact. The vertical line at x = 0 represents no impact on the prediction.

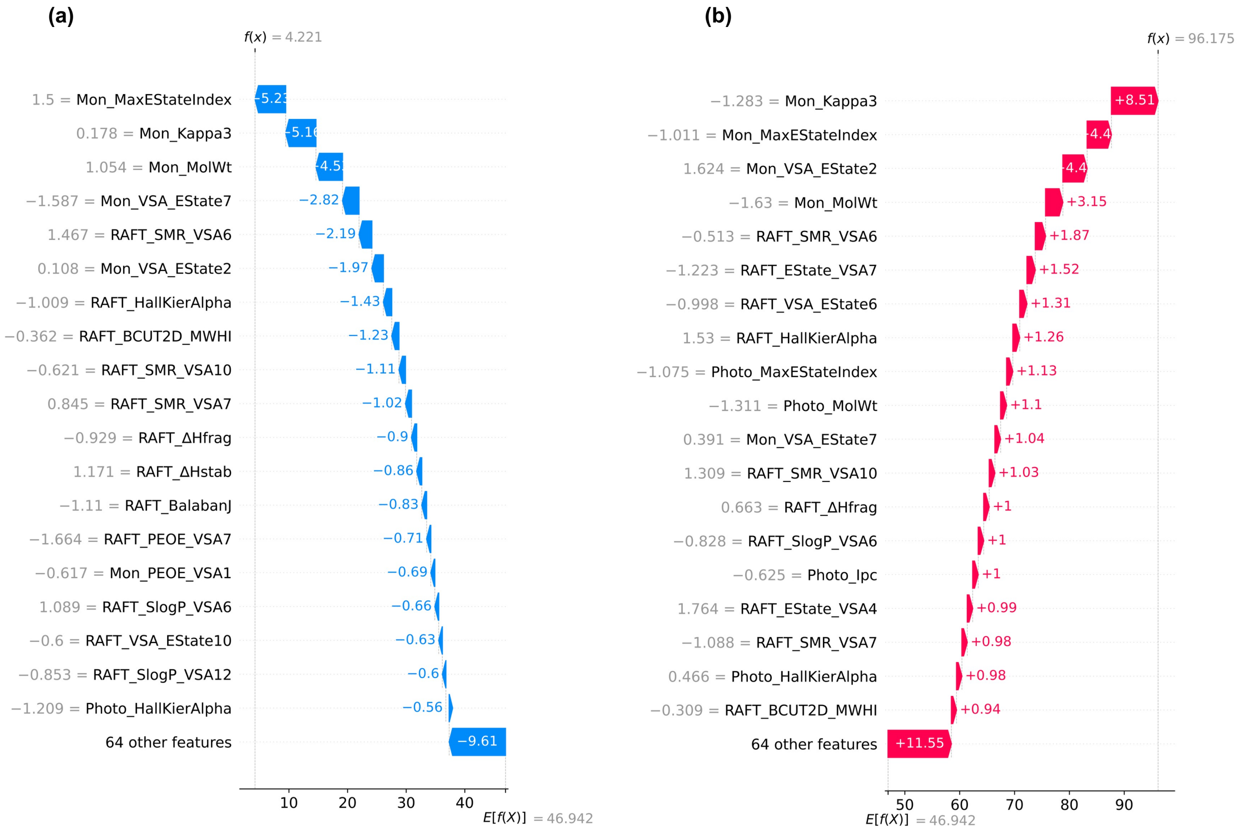

Figure S6. Local SHAP waterfall plots show how different features contribute to PET-RAFT conversion predictions for two typical reactions. (a) A low-conversion example (4% conversion, BzMA with ZnTCPP and DTC2), where bulky monomer descriptors (high Mon_Kappa3, Mon_MolWt) and unfavorable RAFT parameters hindered monomer conversion. (b) A high-conversion example (96% conversion, DMA with ZnTPP and BTPA), where favorable monomer topology (low Mon_Kappa3), low molecular weight, and efficient RAFT fragmentation enthalpy (ΔH_frag) increased the prediction. Positive (blue) and negative (red) bars show additive feature contributions relative to the model baseline of *E*[f(x)] = 46.942.

References

[1] S. Barman, U. Sarkar, *Adv Theory Simul* **2025**, 2401517.

[2] E. H. Krenske, E. I. Izgorodina, M. L. Coote, *ACS Symposium Series* **2006**, *944*, 406.

[3] P. M. Lathrop, Z. Duan, C. Ling, Y. A. Elabd, C. Kravaris, *Processes 2019, Vol. 7, Page 768* **2019**, *7*, 768.

[4] G. Ng, K. Jung, J. Li, C. Wu, L. Zhang, C. Boyer, *Polym Chem* **2021**, *12*, 6548.

[5] Supervised learning — scikit-learn 1.7.1 documentation.
